# Supplementary material for: MAP4Ks inhibition promotes retinal neuron regeneration from Müller glia in adult mice
Source: NPJ Regen Med. 2023 Jul 13;8:36. doi: 10.1038/s41536-023-00310-6 (PMC10344969; doi:10.1038/s41536-023-00310-6)
Supplement: Supplementary file 1 — Supplementary Information [file 41536_2023_310_MOESM1_ESM.pdf]

Supplementary Information

Supplementary Figures and Legends

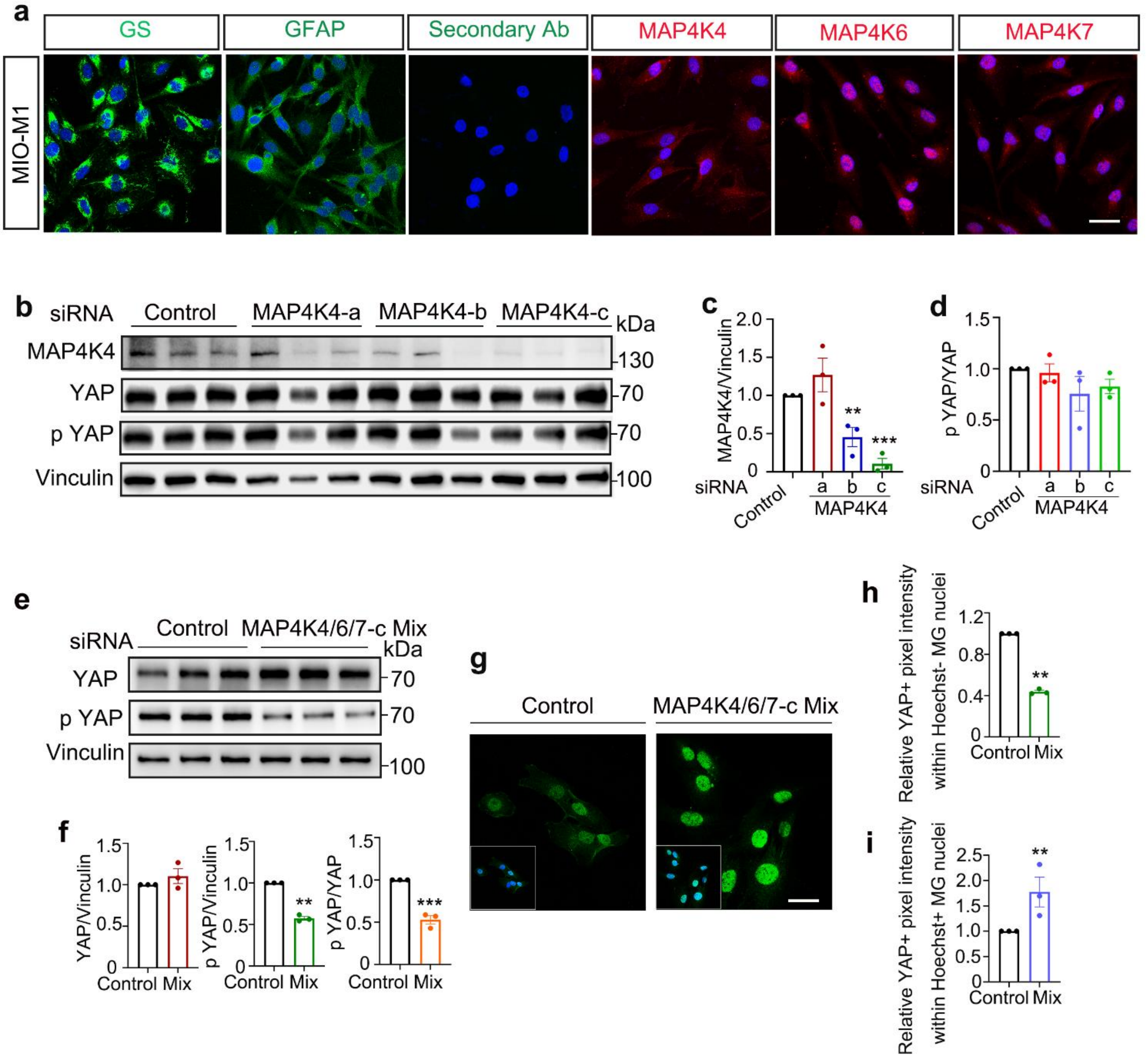

Supplementary Figure 1. MAP4K4/6/7 regulates YAP phosphorylation in MIO-M1. a Immunofluorescence staining of Müller glia marker GS, GFAP, as well as MAP4K4/6/7 in MIO-M1. Immunofluorescence staining of secondary antibody was served as negative control. **b-d** Western blot analysis of MAP4K4, p YAP and YAP (**b**) and quantification (**c,d**; normalized to vinculin) in MIO-M1 treated with MAP4K4 siRNAs. **e, f** Western blot analysis of p YAP and YAP (**e**) and quantification (**f**; normalized to vinculin) in MIO-M1 after control or MAP4K4/6/7 siRNAs treatment. **g** YAP (YAP+; *green*) and Hoechst

(Hoechst+; *blue)* immunofluorescence in MIO-M1 after control or MAP4K4/6/7 siRNAs treatment. **h** Quantification of relative YAP+ pixel intensity in Hoechst- MG regions in **g**. **i** Quantification of relative YAP+ pixel intensity in Hoechst+ MG nuclei in **g**. Scale bars, 20 μm（**a, g**）. For Western blots, levels were given as a.u. ± SEM in comparison with the control group (n = 3 independent pooled samples per group; Student's *t*-test). *p≤0.05, **p≤0.01, ***p≤0.001.

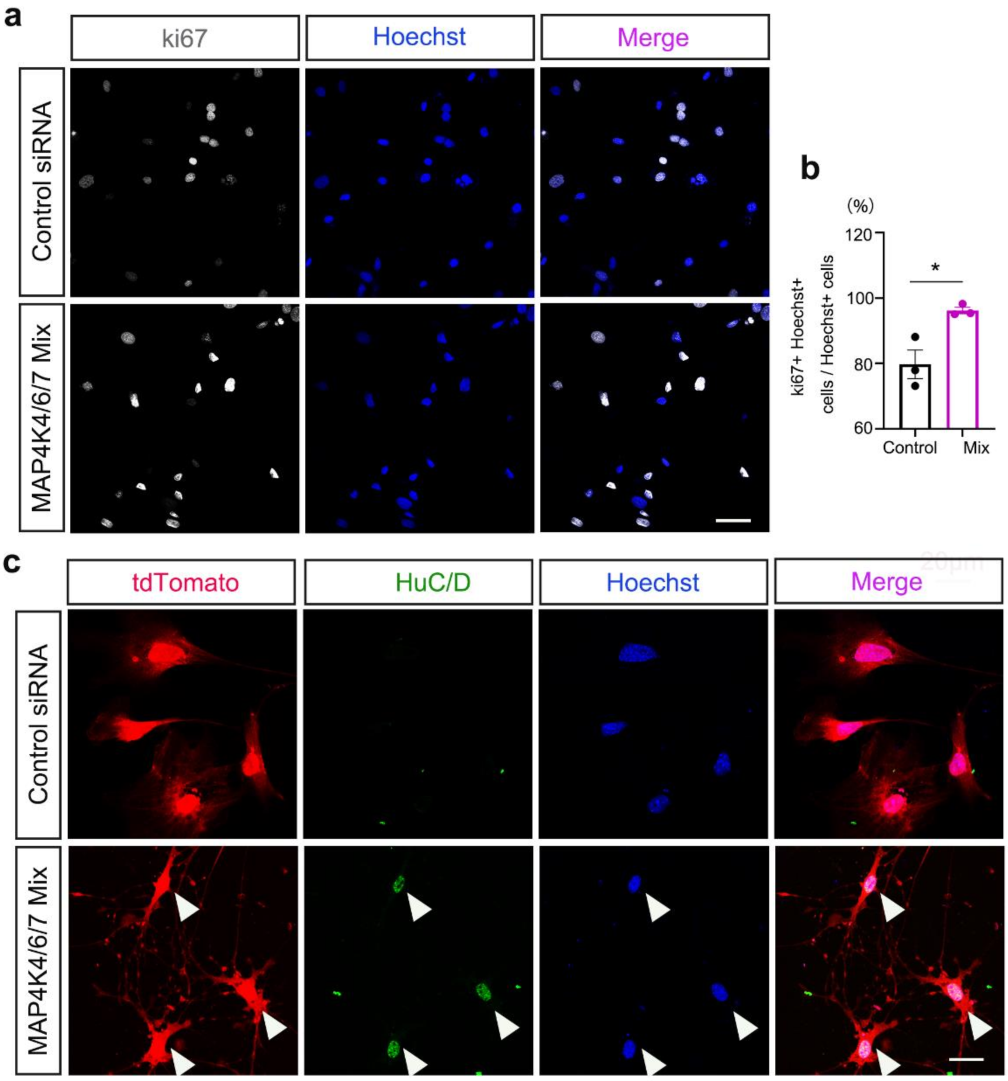

Supplementary Figure 2. MAP4K4/6/7 regulate MG plasticity in cultured cells. a, b Immunofluorescence staining of ki67(**a**) and quantification of the number of ki67+cells(**b**) in MIO-M1cells treated with and without MAP4K4/6/7 siRNAs. **c** Immunofluorescence staining of HuC/D in murine primary MG and cells treated with MAP4K4/6/7 siRNAs. Scale bars, 20 μm (**a, c**). For quantification of ki67+ cells, levels were given as mean ± SEM (n = 3 dishes per group; Student's *t*-test). *$p \leq 0.05$, **$p \leq 0.01$, ***$p \leq 0.001$.

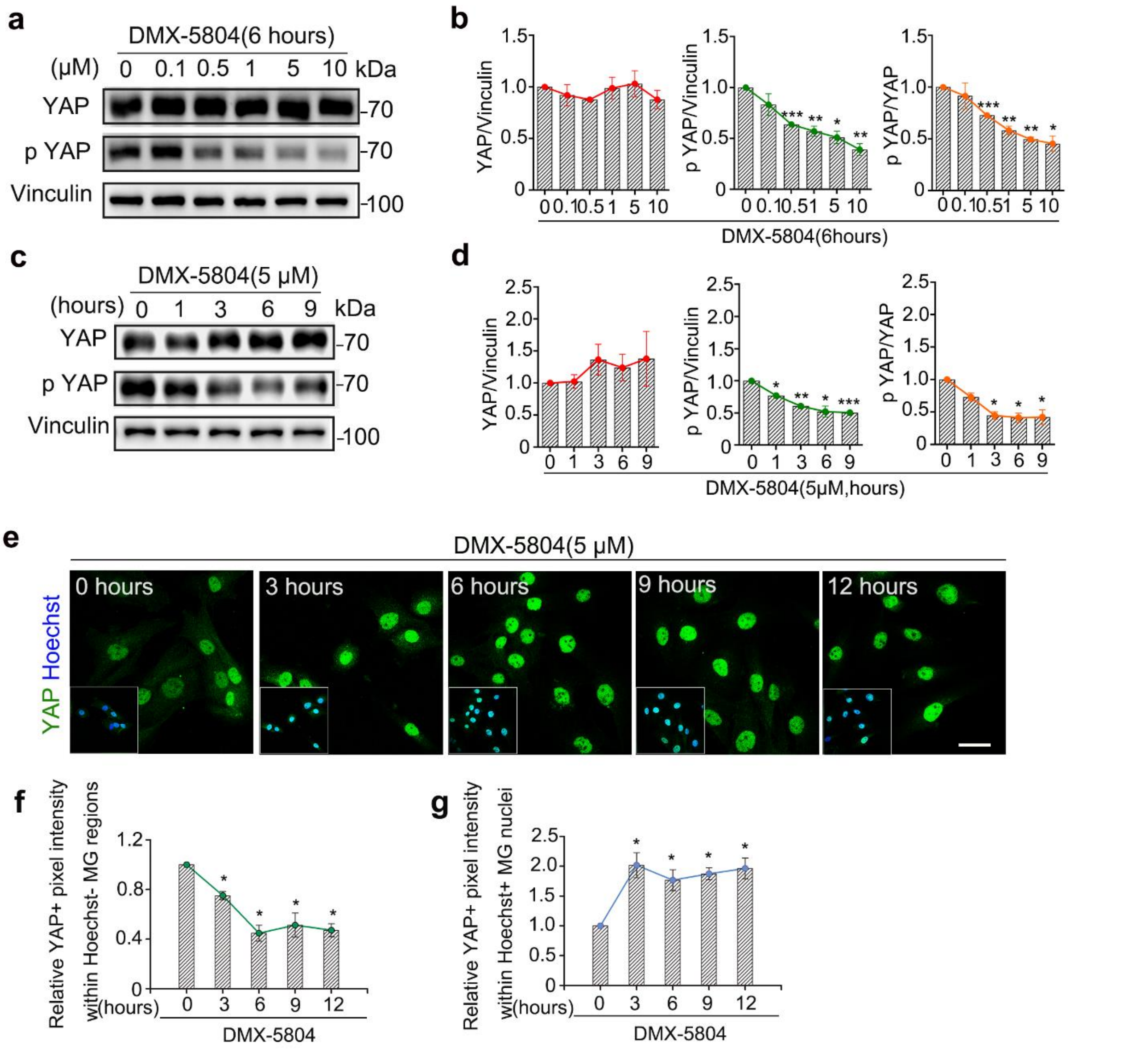

Supplementary Figure 3. MAP4K4/6/7 inhibitor DMX-5804 suppresses YAP phosphorylation in MIO-M1. a, b Western blots (**a**) and quantification (**b**; normalized to vinculin) of p YAP and YAP in MIO-M1 after various concentrations of DMX-5804 treatment. **c. d** Western blots (**c**) and quantification (**d**; normalized to vinculin) of p YAP and YAP in MIO-M1 at indicated time points after 5 μM DMX-5804 treatment. **e** Immunofluorescence staining of YAP in MIO-M1 after 5 μM DMX-5804 treatment. **f** Quantification of relative YAP+ pixel intensity in Hoechst- MG regions in **e**. **g** Quantification of relative YAP+ pixel intensity in Hoechst+ MG nuclei in **e**. Scale bar, 20 μm (**e**). For Western blots, levels were given as a.u. ± SEM relative (n = 3 independent pooled samples per group; Student's *t*-test). For pixel intensity measurements, levels were given as mean ± SEM (n = 3 dishes per group; Student's *t*-test). *p≤0.05, **p≤0.01, ***p≤0.001.

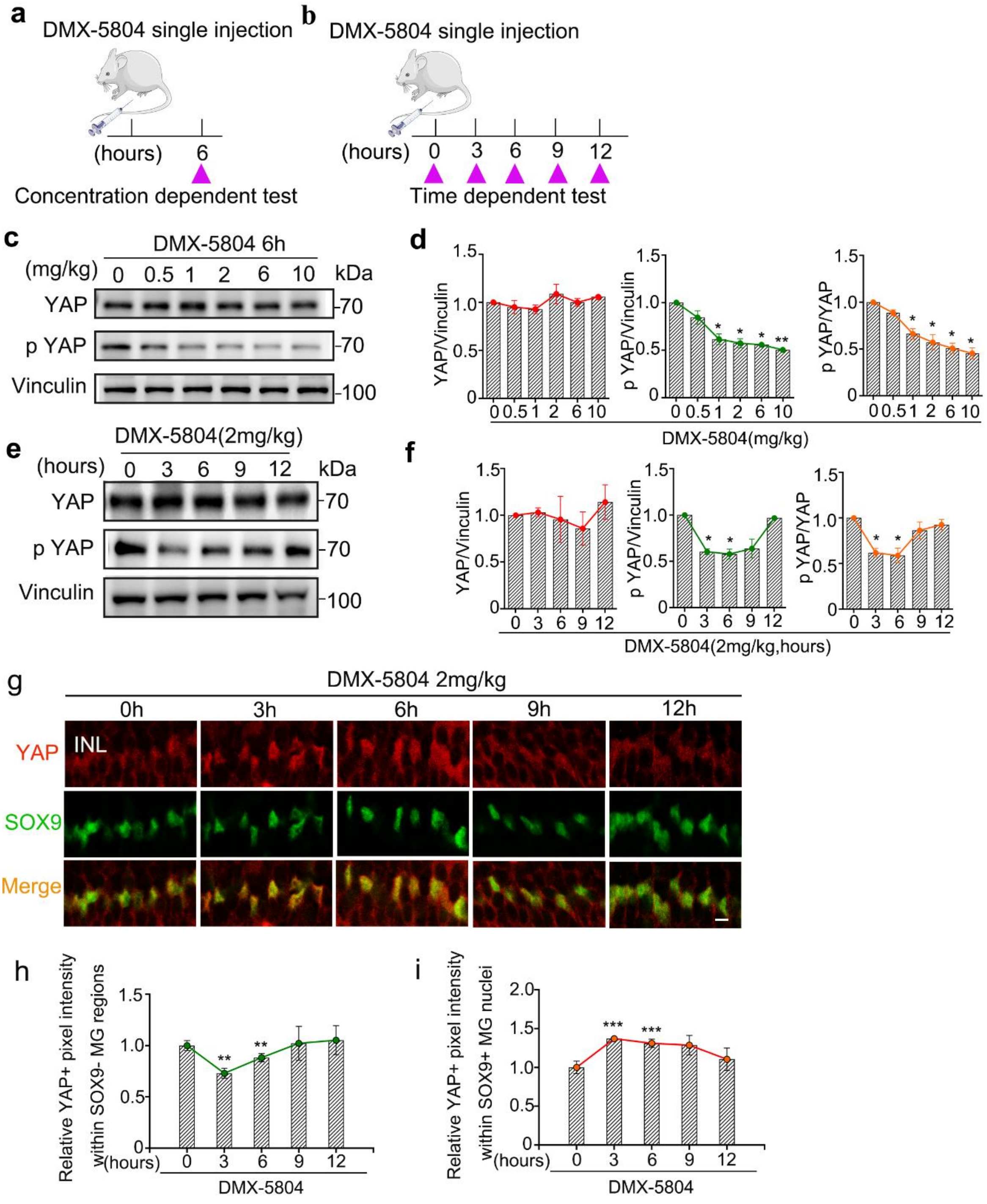

Supplementary Figure 4. MAP4K4/6/7 inhibitor DMX-5804 suppresses YAP phosphorylation in MG of murine retina. a, b Timeline diagram of the experimental procedures used in **c-f**. Single intraperitoneal injection of DMX-5804 was carried out for concentration- **(c)** and time- **(e)** dependent test respectively. The *purple* triangles represent the time points of the sampling. **c, d** Western blots (**c**) and quantification (**d**; normalized to vinculin) of p YAP and YAP after single dose of various concentrations of DMX-5804 injection. **e, f** Western blots (**e**) and quantification (**f**; normalized to vinculin) of p YAP and YAP at indicated time

points after 2 mg/kg DMX-5804 injection. **g** YAP (YAP+, *red*) and SOX9 (SOX9+, *green*) immunofluorescence in mouse retinas after single DMX-5804 injection. **h** Quantification of relative YAP+ pixel intensity in SOX9- MG regions in **g**. **i** Quantification of relative YAP+ pixel intensity in SOX9+ MG nuclei in **g**. Scale bar, 5 µm (**g**). For western blots, levels were given as a.u. ± SEM relative no-treatment (NT) (4 samples per pool; n = 3 independent pooled samples per group; Student's *t* test). For pixel intensity measurements, levels are given as mean ± SEM (n = 6 mice per group; Student's *t*-test). **p*≤0.05, ***p*≤0.01, ****p*≤0.001.

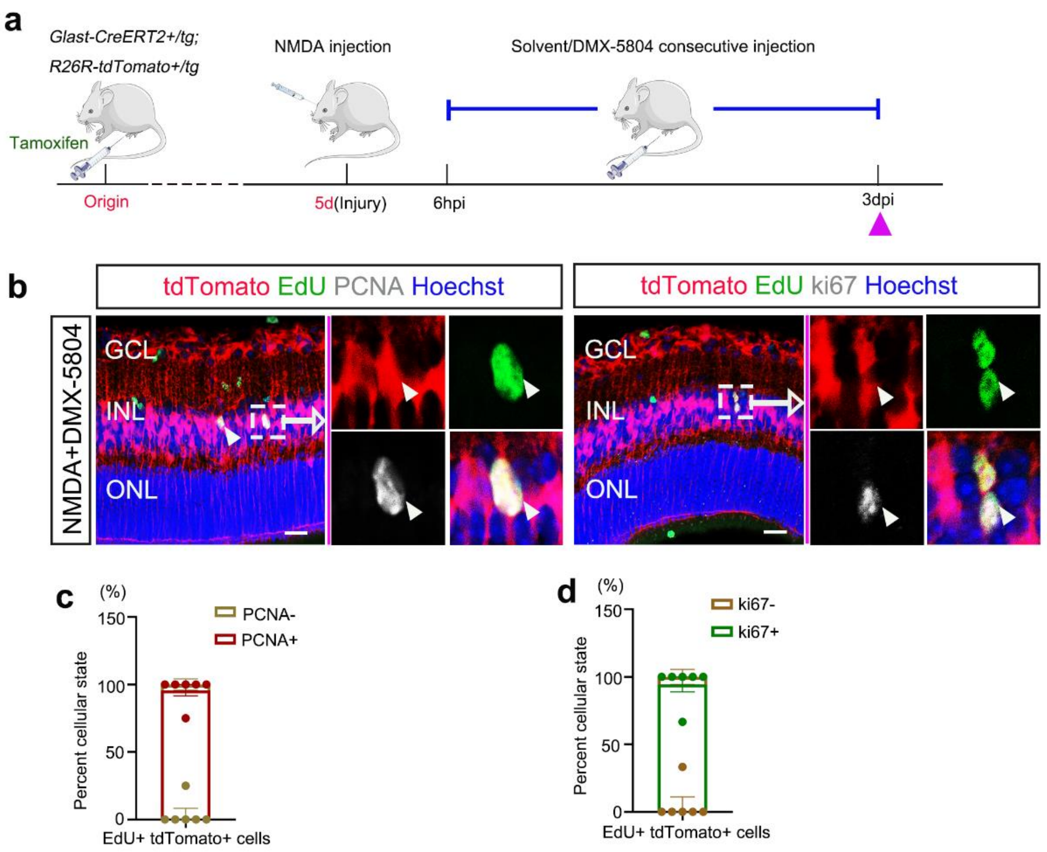

Supplementary Figure 5. The expression of the markers of proliferation in MG of the NMDA-injured murine retina with or without DMX-5804 treatment. a Timeline diagram of the experimental procedures used in **b-d**. Glast-CreERT2+/tg;ROSA26R-tdTomato+/tg mice were intraperitoneally injected with Tamoxifen for 5 consecutive days. Then NMDA was intraocularly administered. DMX-5804 was injected every 6 h intraperitoneally from 6 hpi until 3 dpi after NMDA injection. The *purple* triangles represent the time points of the sampling. **b** EdU labeling (*green*) and PCNA or ki67 immunofluorescence (*gray*) on retinal sections after NMDA/DMX-5804 treatment. **c** Quantification of the percentage of cells positive for both PCNA+ cells in EdU+tdTomato+ cells in **b**. **d** Quantification of the percentage of cells positive for both ki67+ cells in EdU+tdTomato+ cells in **b**. Scale bars, 20 μm (**b**). For quantification of PCNA+ or ki67+ cells, levels were given as mean ± SEM (n = 6 mice per group; Student's *t*-test). *$p \leq 0.05$, **$p \leq 0.01$, ***$p \leq 0.001$.

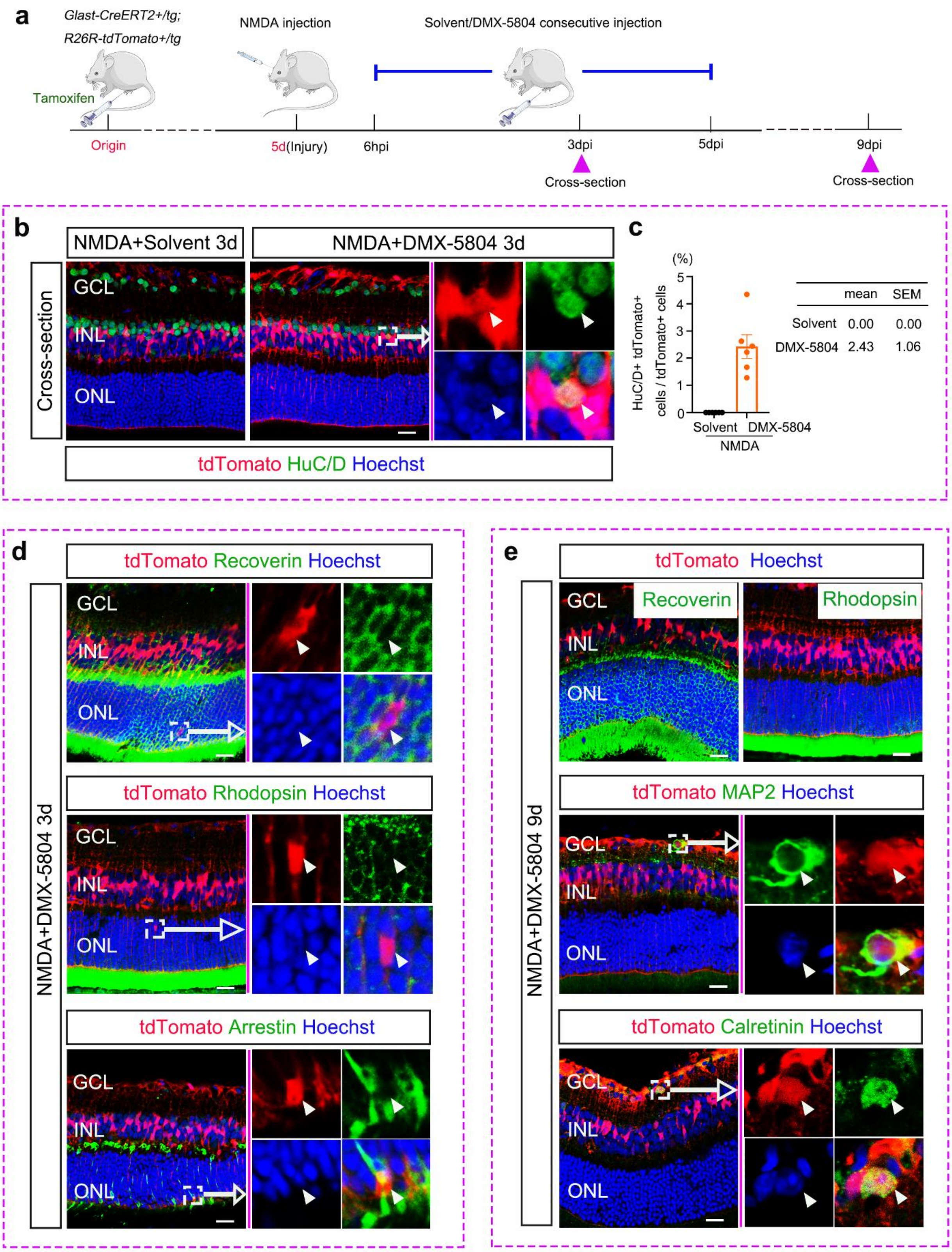

Supplementary Figure 6. The expression of neuronal markers in MG-derived cells of the NMDA-injured murine retina with or without DMX-5804 treatment. a Timeline diagram of the experimental procedures used in **b-e**. Glast-CreERT2+/tg;ROSA26R-tdTomato+/tg mice were intraperitoneally injected

with Tamoxifen for 5 consecutive days. Then NMDA was intraocularly administered. DMX-5804 was injected every 6 h intraperitoneally from 6 hpi until 5 dpi after NMDA injection. The *purple* triangles represent the time points of the sampling. **b** HuC/D (*green*) immunofluorescence in the NMDA-injured retinas after 3 days of DMX-5804 treatment. **c** Quantification of the percentage of HuC/D+ cells in tdTomato+ cells in **b**. **d** Recoverin (*green*), Rhodopsin (*green*) and Arrestin (*green*) immunofluorescence in the NMDA-injured retinas after 3 days of DMX-5804 treatment. **e** Recoverin (*green*), Rhodopsin (*green*), MAP2 (*green*) and Calretinin (*green*) immunofluorescence in the NMDA-injured retinas after 9 days of DMX-5804 treatment. Scale bars, 20 μm (**b, d, e**). For quantification of HuC/D+ cells, levels were given as mean ± SEM (n = 9 mice per group; Student's *t*-test). **p*≤0.05, ***p*≤0.01, ****p*≤0.001.

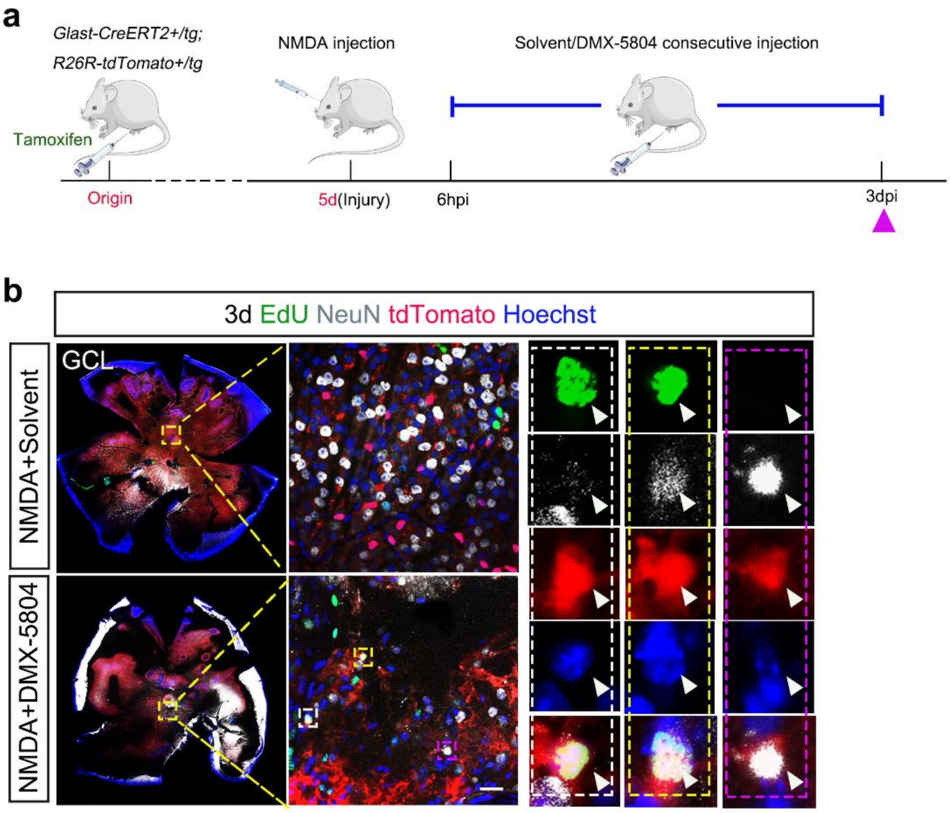

Supplementary Figure 7. MAP4K4/6/7 inhibitor DMX-5804 promotes the generation of MG-derived neurons. a Timeline diagram of the experimental procedures used in **b**. Glast-CreERT2+/tg;ROSA26R-tdTomato+/tg mice were intraperitoneally injected with Tamoxifen for 5 consecutive days. Then NMDA was intraocularly administered. DMX-5804 was injected every 6 h intraperitoneally from 6 hpi until 3 dpi after NMDA injection. The *purple* triangles represent the time points of the sampling. **b** EdU labeling (*gray*) and NeuN (*green*) immunofluorescence in the NMDA-injured retinas after DMX-5804 treatment. Scale bar, 20 μm (**b**).

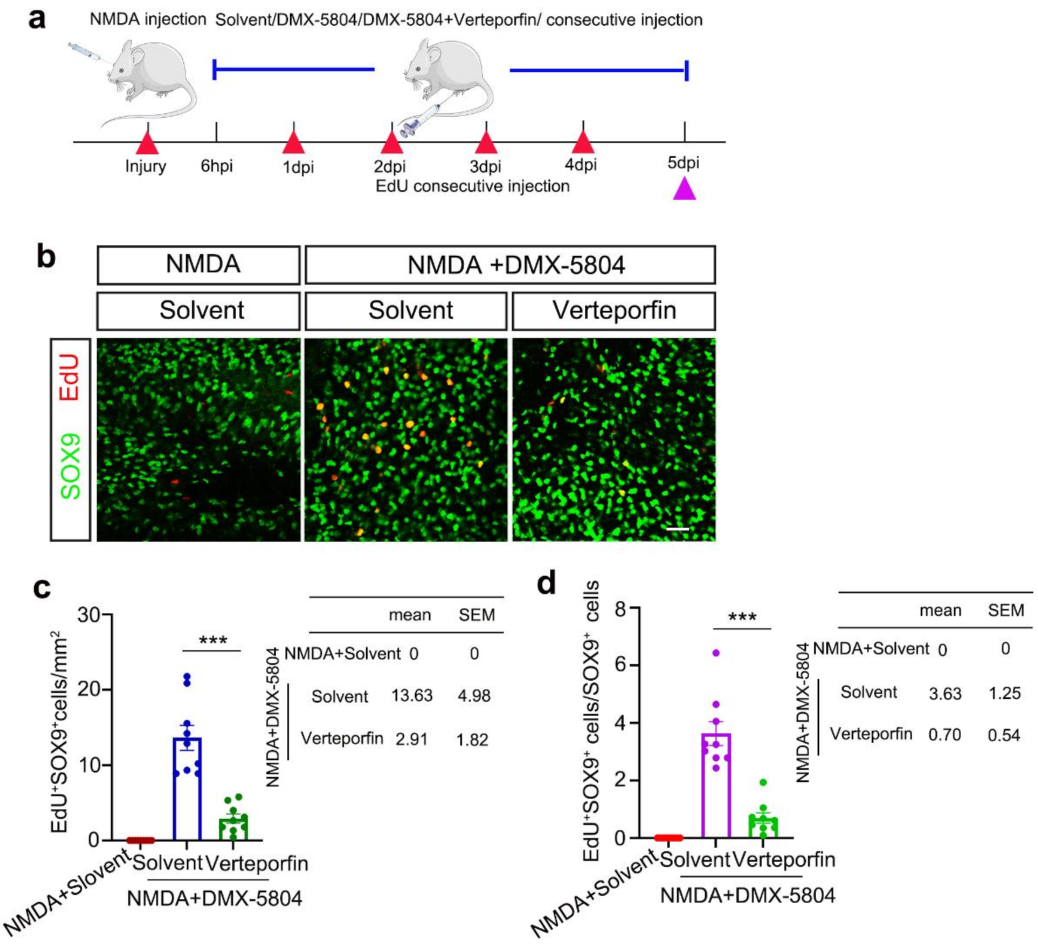

Supplementary Figure 8. NMDA/DMX-5804 induced MG proliferation is absent after verteporfin treatment. a Timeline diagram of the experimental procedures used in **b-d**. Wild type mice were intravitreally injected with NMDA. DMX-5804/solvent or DMX-5804/verteporfin were delivered every 6 h intraperitoneally from 6 hpi to 5 dpi. EdU was injected intraperitoneally every 24 h until 5 days after NMDA injury. **b** EdU labeling (EdU+; *red*) and SOX9 immunofluorescence (SOX9+; *green*) on whole flat-mounted retinas 5 days after NMDA injection and DMX-5804/solvent or DMX-5804/verteporfin treatment. **c** Quantification of the number of cells positive for both EdU labeling (EdU+; *re*d) and SOX9 immunofluorescence (SOX9+; *green*) per mm^2 in **b**. **d** Quantification of the percentage of EdU+ SOX9+ cells in SOX9+ cell in **b**. Scale bar, 30 μm (**b**). For quantification of EdU+ SOX9+ cells, levels were given as mean ± SEM (n = 9 mice per group; Student's *t*-test). **p*≤0.05, ***p*≤0.01, ****p*≤0.001.

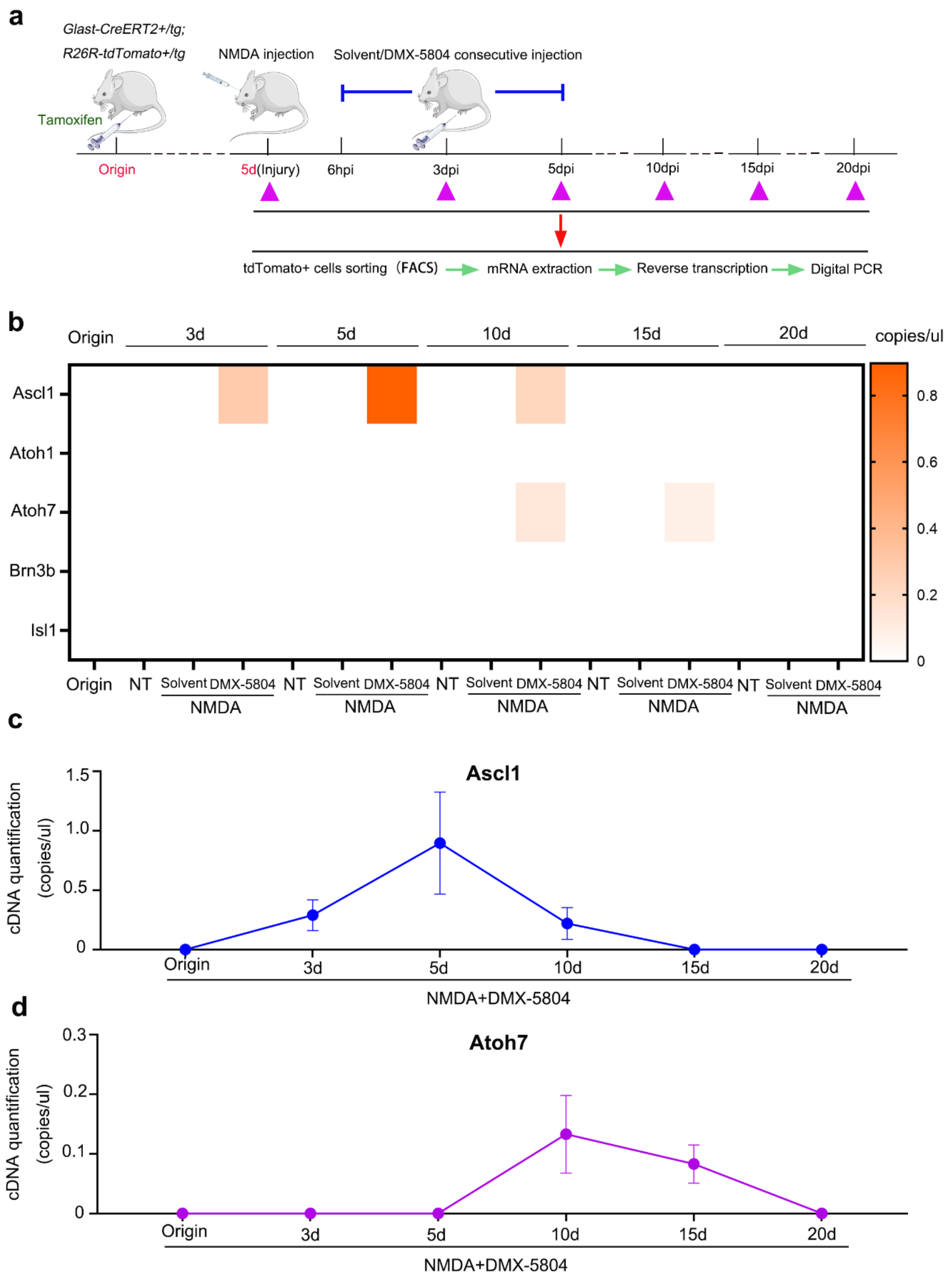

Supplementary Figure 9. The expression of proneural transcription factors in MG after NMDA/DMX-5804 induction. a Timeline diagram of the experimental procedures used in **b-d**. Glast-CreERT2+/tg;ROSA26R-tdTomato+/tg mice were intraperitoneally injected with Tamoxifen for 5 consecutive days. Then NMDA was intraocularly administered. DMX-5804 was injected every 6 h

intraperitoneally from 6 hpi until 5 dpi after NMDA injection. Then, 0,3,5,10,15 and 20 days after NMDA injection, mice were sacrificed and retinal samples were analyzed. The *purple* triangles represent the time points of the sampling. **b** Heatmap of several key transcription factors in isolated MGs after NMDA/Solvent or NMDA/DMX-5804 treatment. **c, d** Quantification of the mRNA levels of Ascl1 and Atoh7 in MG.

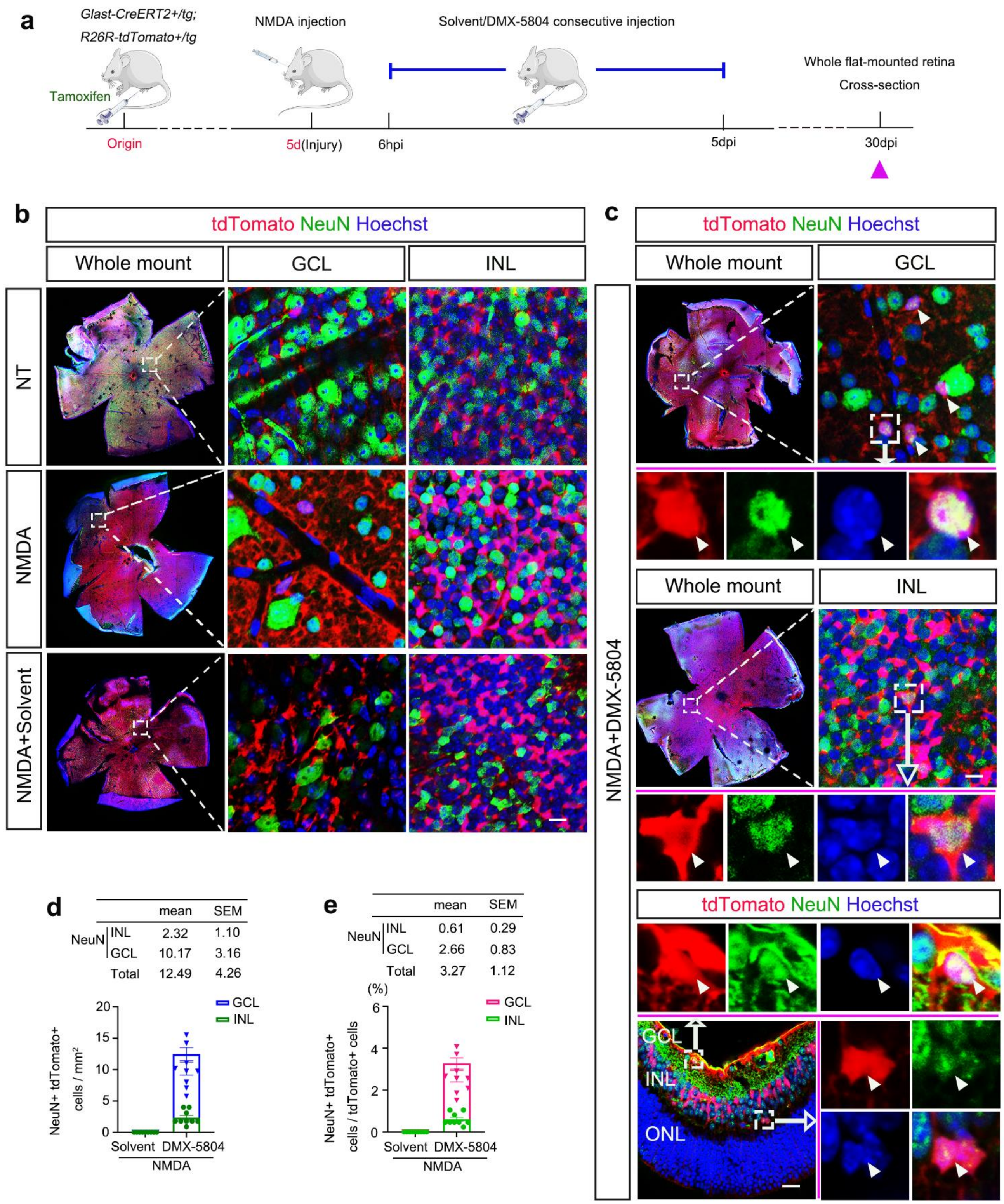

Supplementary Figure 10. The assessment of MG-to-neuron conversion efficiency with a long-term tracing after DMX-5804 treatment. a Timeline diagram of the experimental procedures used in **b-d**. Glast-CreERT2+/tg;ROSA26R-tdTomato+/tg mice were intraperitoneally injected with Tamoxifen for 5 consecutive days. Then NMDA was intraocularly administered. DMX-5804 was injected every 6 h

intraperitoneally from 6 hpi until 5 dpi after NMDA injection. Thirty (30) days after NMDA injection, mice were sacrificed and retinal samples were analyzed. The *purple* triangles represent the time points of the sampling. **b, c** NeuN (*green*) immunofluorescence on whole flat-mounted NMDA-injured retinas after Solvent or DMX-5804 treatment(**b, c**) and NeuN (green) immunofluorescence on retinal sections after DMX-5804 treatment(**c**). **d** Quantification of the number of NeuN+tdTomato+, per mm^2 in **b** and **c**. **e** Quantification of the percentage of NeuN+tdTomato+ cells in tdTomato+ cells in **b** and **c**. Scale bars, 20 μm (**b, c**). For quantification of NeuN+ tdTomato+ cells, levels were given as mean ± SEM (n = 9 mice per group).

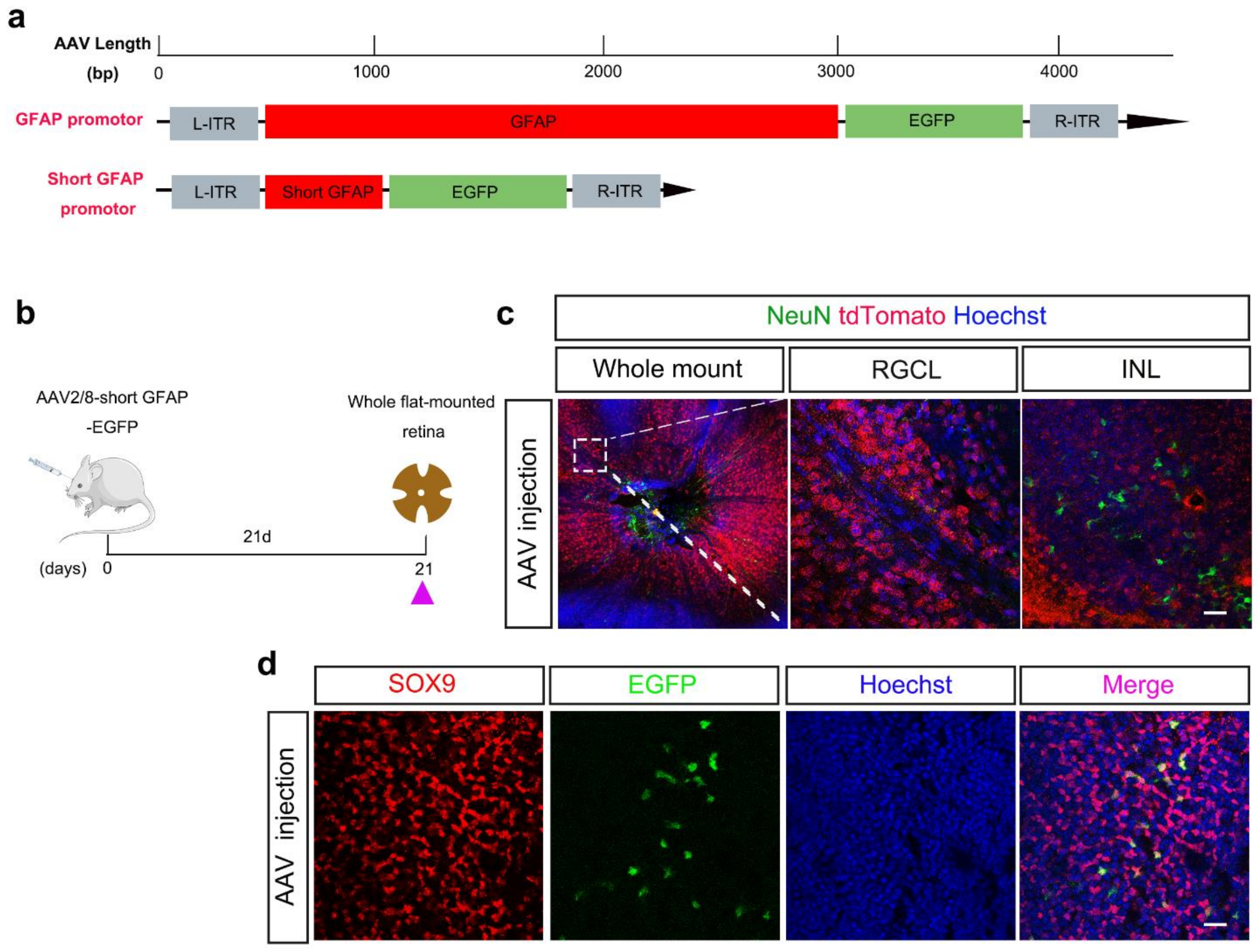

Supplementary Figure 11. The specificities of AAV tracing systems used in this study. a Diagram of the difference between GFAP and Short GFAP promotor in AAV vectors. **b** Timeline diagram of the experimental procedures used in **c** and **d**. Wildtype mice were intravitreally injected with pAAV-short GFAP-MCS-EGFP-3FLAG. Mice were sacrificed on 21st day and whole flat-mounted retinas were prepared. The *purple* triangles represent the time points of the sampling. **c** NeuN (*green*) immunofluorescence on whole flat-mounted retinas three weeks after infection with pAAV-short GFAP-MCS-EGFP-3FLAG. **d** SOX9 (*red*) immunofluorescence on whole flat-mounted retinas three weeks after infection with pAAV-short GFAP-MCS-EGFP-3FLAG. Scale bars, 20 μm (**c, d**).

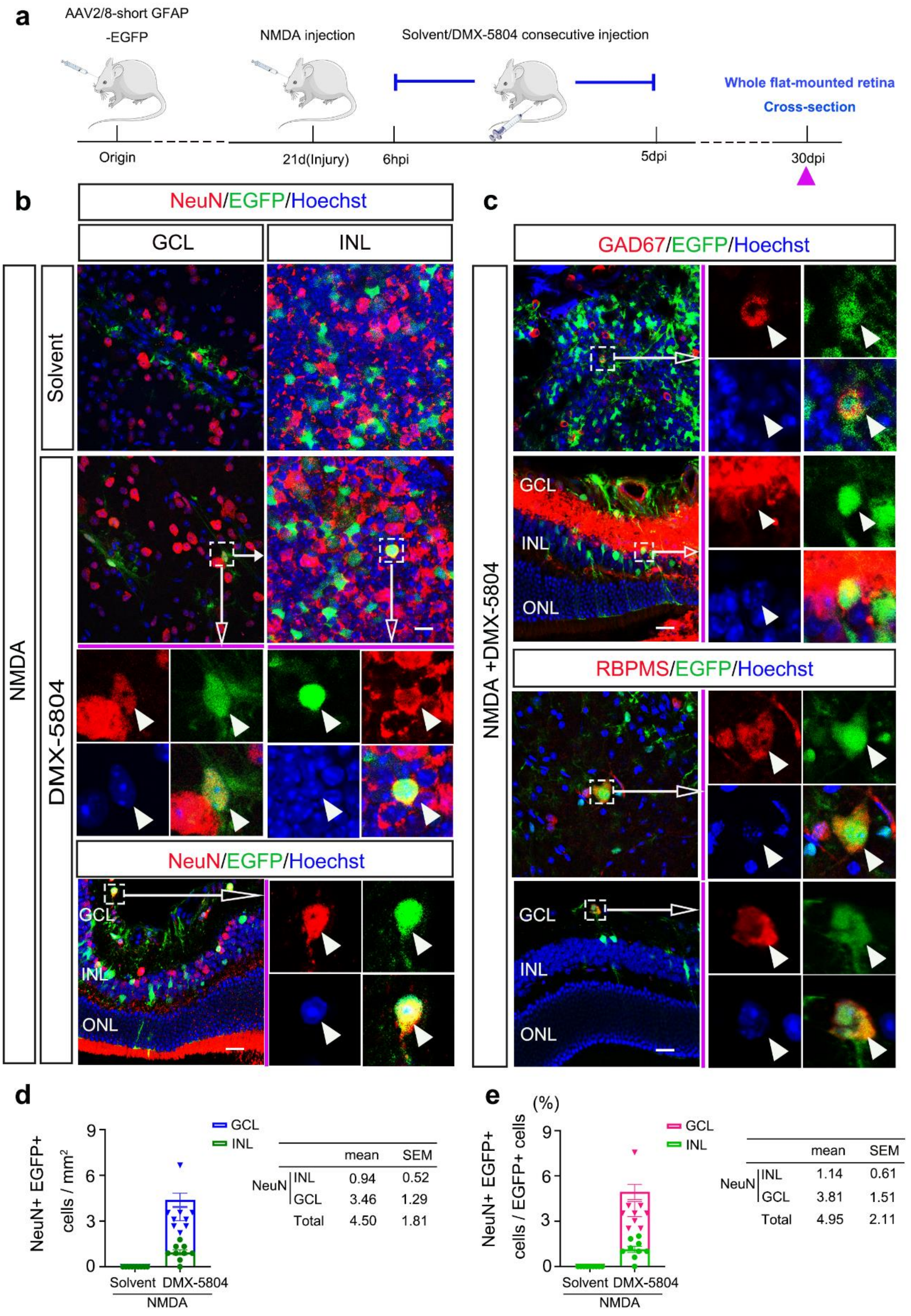

		mean	SEM
NeuN	INL	0.94	0.52
	GCL	3.46	1.29
	Total	4.50	1.81

		mean	SEM
NeuN	INL	1.14	0.61
	GCL	3.81	1.51
	Total	4.95	2.11

Supplementary Figure 12. Long-term tracing of MG fate after NMDA/DMX-5804 treatment by AAV tracing system. a Timeline diagram of the experimental procedures used in **b-e**. Wildtype mice were intravitreally injected with pAAV-short GFAP-MCS-EGFP-3FLAG. NMDA was intraocularly administered 3

wk later. Then DMX-5804 was injected every 6 h intraperitoneally from 6 hpi until 5 dpi after NMDA injection. Thirty (30) days after NMDA injection, mice were sacrificed and the retinas were analyzed. The *purple* triangles represent the time points of the sampling. **b** NeuN (NeuN+; *red*) immunofluorescence on whole flat-mounted NMDA-injured retinas retinal sections infected with pAAV-short GFAP-MCS-EGFP-3FLAG after solvent or DMX-5804 treatment. **c** GABAergic cell marker GAD67 (GAD67+; *red*) or retinal ganglion cell marker (RBPMS+; *red*) immunofluorescence on whole flat-mounted retinas and retinal sections infected with pAAV-short GFAP-MCS-EGFP-3FLAG after NMDA/DMX-5804 treatment. **d** Quantification of the number of NeuN+GFP+, GAD67+GFP+ cells or RBPMS+GFP+ cells per mm^2 in **b** and **c**. **e** Quantification of the percentage of NeuN+GFP+, GAD67+GFP+ cells or RBPMS+GFP+ cells in GFP+ cells in **b** and **c**. Scale bars, 20 μm (**b, c**). For quantification of NeuN+ GFP+ cells, levels were given as mean ± SEM (n = 9 mice per group).

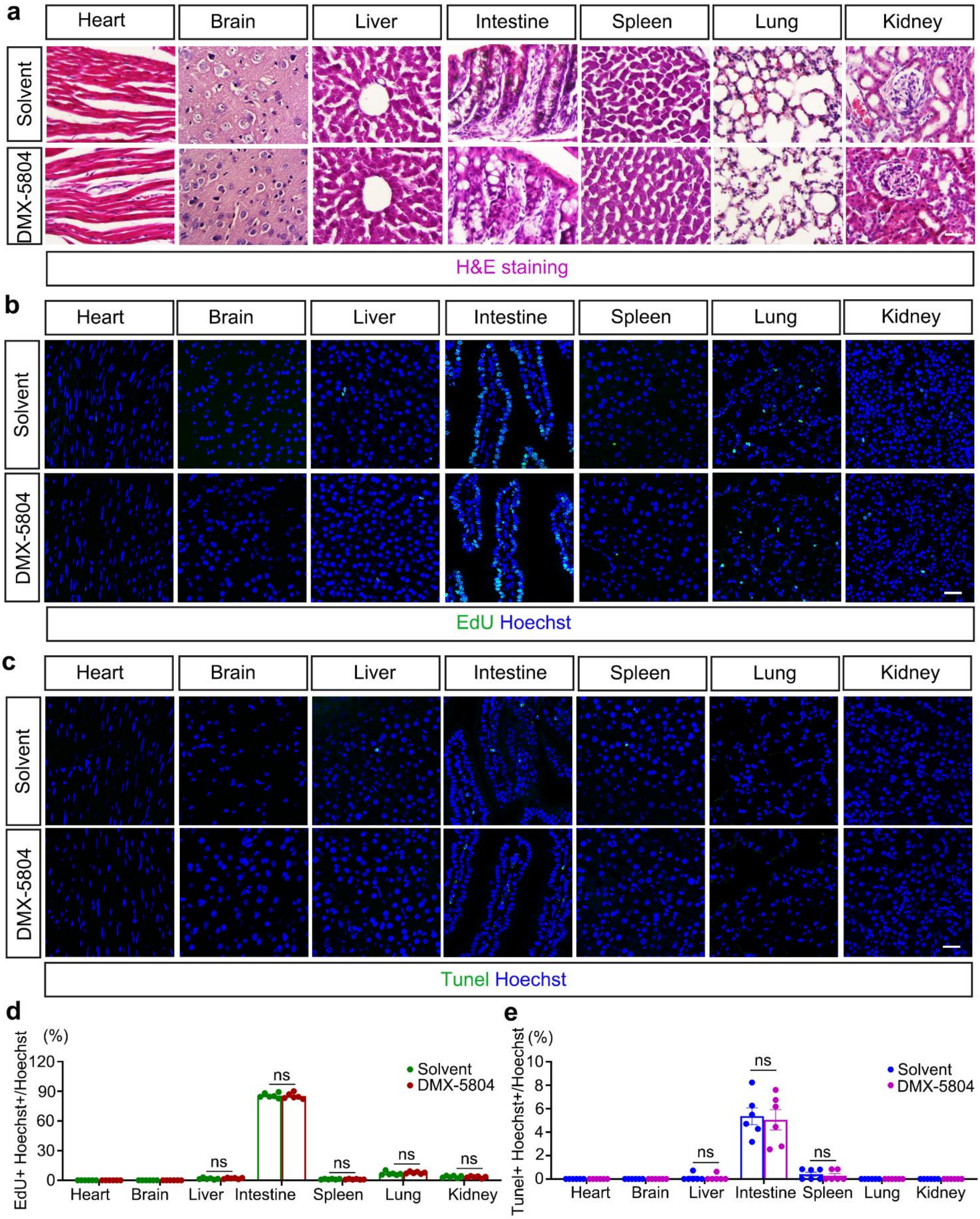

Supplementary Figure 13. The assessment of systemic side effect of DMX-5804. **a** H&E staining of slides from various organs after DMX-5804 treatment. **b** EdU labeling (EdU+; green) in various organs after DMX-5804 treatment. **c** TUNEL staining (TUNEL+; green) in various organs after DMX-5804 treatment. **c** Quantification of the percentage of EdU+Hoechst+ cells in Hoechst+ cells presented in **b**. **e**

Quantification of the percentage of TUNEL+Hoechst+ cells in Hoechst+ cells presented in **c**. Scale bars, 35 μm (**a**) or 25 μm (**b, c**). For quantification of EdU+ or TUNEL+ cells, results were given as mean ± SEM (n = 9 mice per group; Student's *t*-test). *$p \leq 0.05$, **$p \leq 0.01$, ***$p \leq 0.001$.

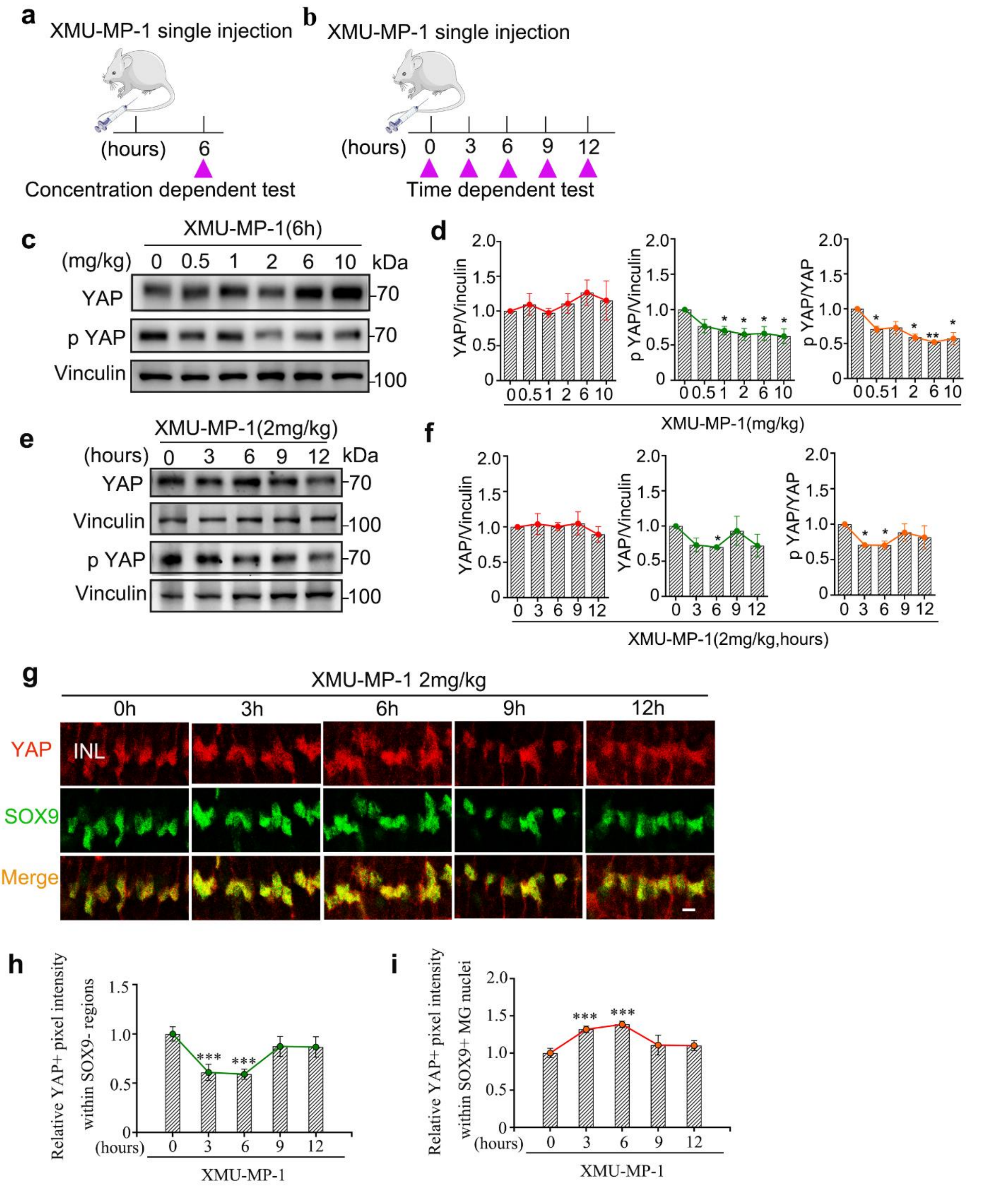

Supplementary Figure 14. Canonical Hippo pathway inhibitor XMU-MP-1 suppresses YAP phosphorylation in MG of murine retina. a, b Timeline diagram of the experimental procedures used in **c-f**. Single intraperitoneal injection of XMU-MP-1 was carried out for concentration- (**c**) and time- (**e**) dependent test, respectively. The *purple* triangles represent the time points of the sampling. **c, d** Western blots (**c**) and quantification (**d**; normalized to vinculin) of p YAP and YAP at 6 h after single dose of various

concentrations of XMU-MP-1 injection. **e, f** Western blots (**e**) and quantification (**f**; normalized to vinculin) of p YAP and YAP at indicated time points after single 2 mg/kg XMU-MP-1 injection. **g** YAP (YAP+; *red*) and SOX9 (SOX9+; *green*) immunofluorescence on retinal sections at indicated time points after single 2 mg/kg XMU-MP-1 injection. **h** Quantification of relative YAP+ pixel intensity in SOX9- MG regions in **g**. **i** Quantification of relative YAP+ pixel intensity in SOX9+ MG nuclei in **g**. Scale bar, 5 μm (**g**). For Western blots, levels were given as a.u. ± SEM relative no-treatment (NT) (4 samples per pool; n = 3 independent pooled samples per group; Student's *t*-test). For pixel intensity measurements, levels were given as mean ± SEM (n = 6 mice per group; Student's *t*-test). *p≤0.05, **p≤0.01, ***p≤0.001.

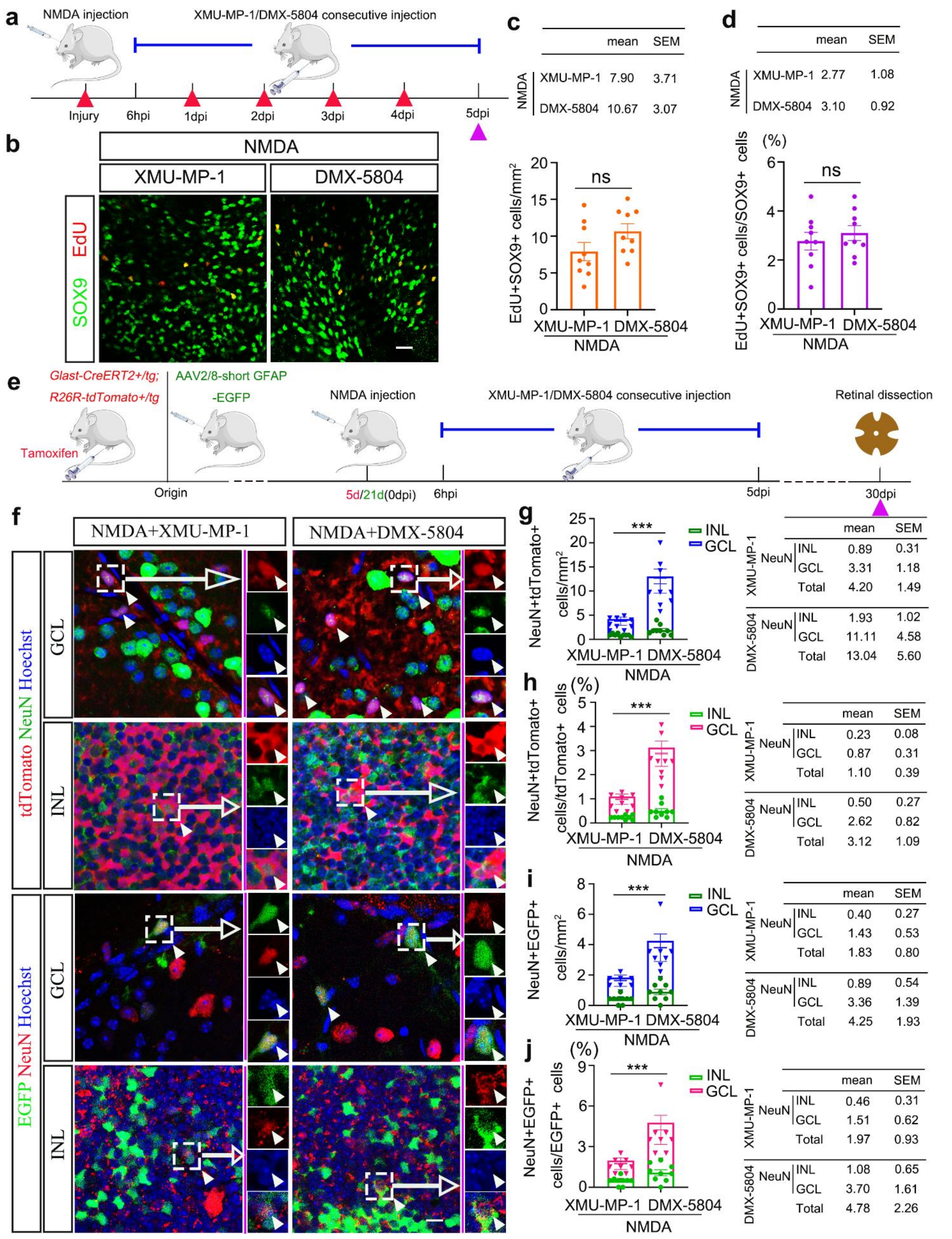

Supplementary Figure 15. The comparison of abilities of XMU-MP-1 and DMX-5804 to promote MG proliferation and trans-differentiation. **a** Timeline diagram of the experimental procedures used in **b-d**.

Wild type mice were intravitreally injected with NMDA first. XMU-MP-1 or DMX-5804 was injected starting from 6 hpi until 5 dpi with injections every 6 h. EdU was injected intraperitoneally every 24 hours. Mice were sacrificed 5 days later. The red triangles represent the time points of the EdU injection and the *purple* triangles represent the time points of the sampling. **b** EdU labeling (Edu+; *red*) and SOX9 (SOX9+; *green*) immunofluorescence on whole flat-mounted retinas 5 days after NMDA injection and consecutive XMU-MP-1 or DMX-5804 treatment. **c** Quantification of the number of EdU+SOX9+ cells on whole flat-mounted retinas 5 days after NMDA injection and consecutive XMU-MP-1 or DMX-5804 treatment. **d** Quantification of the percentage of EdU+ SOX9+ cells in SOX9+ cells on whole flat-mounted retinas 5 days after NMDA injection and consecutive XMU-MP-1 or DMX-5804 treatment. **e** Timeline diagram of the experimental procedures used in **f-j**. Two systems were used. One is Glast-CreERT2+/tg;ROSA26R-tdTomato+/tg mice were intraperitoneally injected with Tamoxifen, and the other is wild type mice were intravitreally injected with pAAV-short GFAP-MCS-EGFP-3FLAG. For Glast-CreERT2+/tg;ROSA26R-tdTomato+/tg mice which were injected with Tamoxifen, NMDA was administered into the vitreous 5 days later. For wild type mice, which were intravitreally injected with pAAV-short GFAP-MCS-EGFP-3FLAG, NMDA was administered into the vitreous 3 wk later. XMU-MP-1 or DMX-5804 was firstly injected starting from 6 hpi until 5 dpi with injections every 6 h. The *purple* triangles represent the time points of the sampling. **f** TdTomato and NeuN (NeuN+; *green*) immunofluorescence or EGFP and NeuN (NeuN+; *red*) immunofluorescence on whole flat-mounted retinas after NMDA injection and consecutive XMU-MP-1 or DMX-5804 treatment. **g** Quantification of the number of tdTomato+NeuN+ cells on whole flat-mounted retinas 30 days after NMDA injection and consecutive XMU-MP-1 or DMX-5804 treatment. **h** Quantification of the percentage of tdTomato+NeuN+ cells in tdTomato+ cells on whole flat-mounted retinas 5 days after NMDA injection and consecutive XMU-MP-1 or DMX-5804 treatment. **i** Quantification of the number of EGFP+NeuN+ cells on whole flat-mounted retinas 30 days after NMDA injection and consecutive XMU-MP-1 or DMX-5804 treatment. **j** Quantification of the percentage of EGFP+NeuN+ cells in EGFP+ cells on whole flat-mounted retinas 5 days after NMDA injection and consecutive XMU-MP-1 or DMX-5804 treatment. Scale bars, 20 µm (**b, f**). For quantification of EdU+ SOX9+, tdTomato+NeuN+ or EGFP+NeuN+ cells, levels were given as mean ± SEM (n = 9 mice per group; Student's *t*-test). *$p \leq 0.05$, **$p \leq 0.01$, ***$p \leq 0.001$.

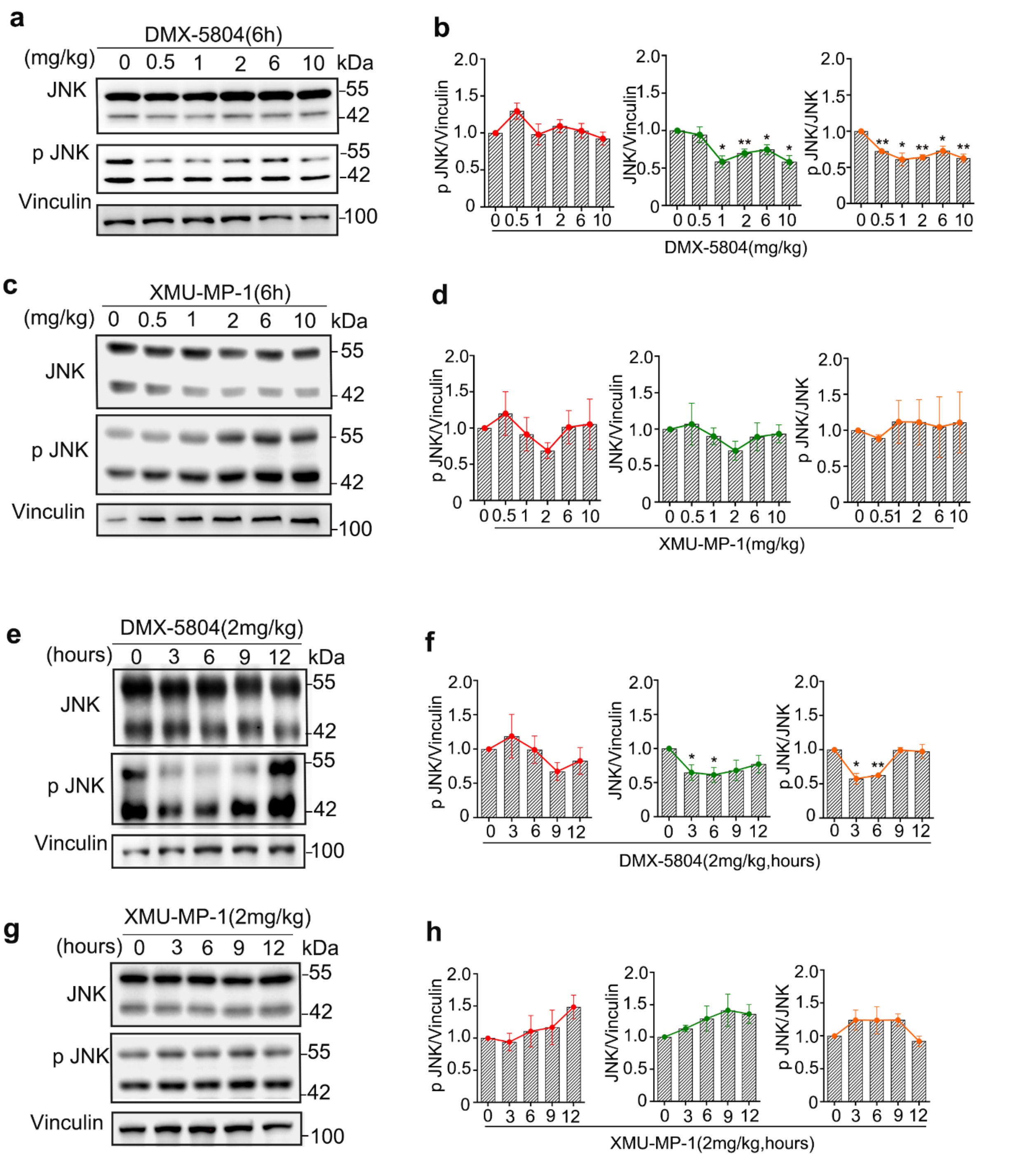

Supplementary Figure 16. DMX-5804 but not XMU-MP-1 regulates JNK pathway activities in murine retina. a, b Western blots (**a**) and quantification (**b**; normalized to vinculin) of p JNK and JNK in murine retinas at 6 h after a single dose of various concentrations of DMX-5804 injection. **c, d** Western blots (**c**) and quantification (**d**; normalized to vinculin) of p JNK and JNK in murine retinas at 6 h after a single dose of various concentrations of XMU-MP-1 injection. **e, f** Western blots (**e**) and quantification (**f**; normalized to vinculin) of p JNK and JNK at indicated time points after a single 2 mg/kg DMX-5804 injection. **g, h**

Western blots (**g**) and quantification (**h**; normalized to vinculin) of p JNK and JNK at indicated time points after a single 2 mg/kg XMU-MP-1 injection. For Western blots, levels were given as a.u. ± SEM relative no-treatment (NT) (4 samples per pool; n = 3 independent pooled samples per group; Student's *t*-test). *$p \leq 0.05$, **$p \leq 0.01$, ***$p \leq 0.001$.

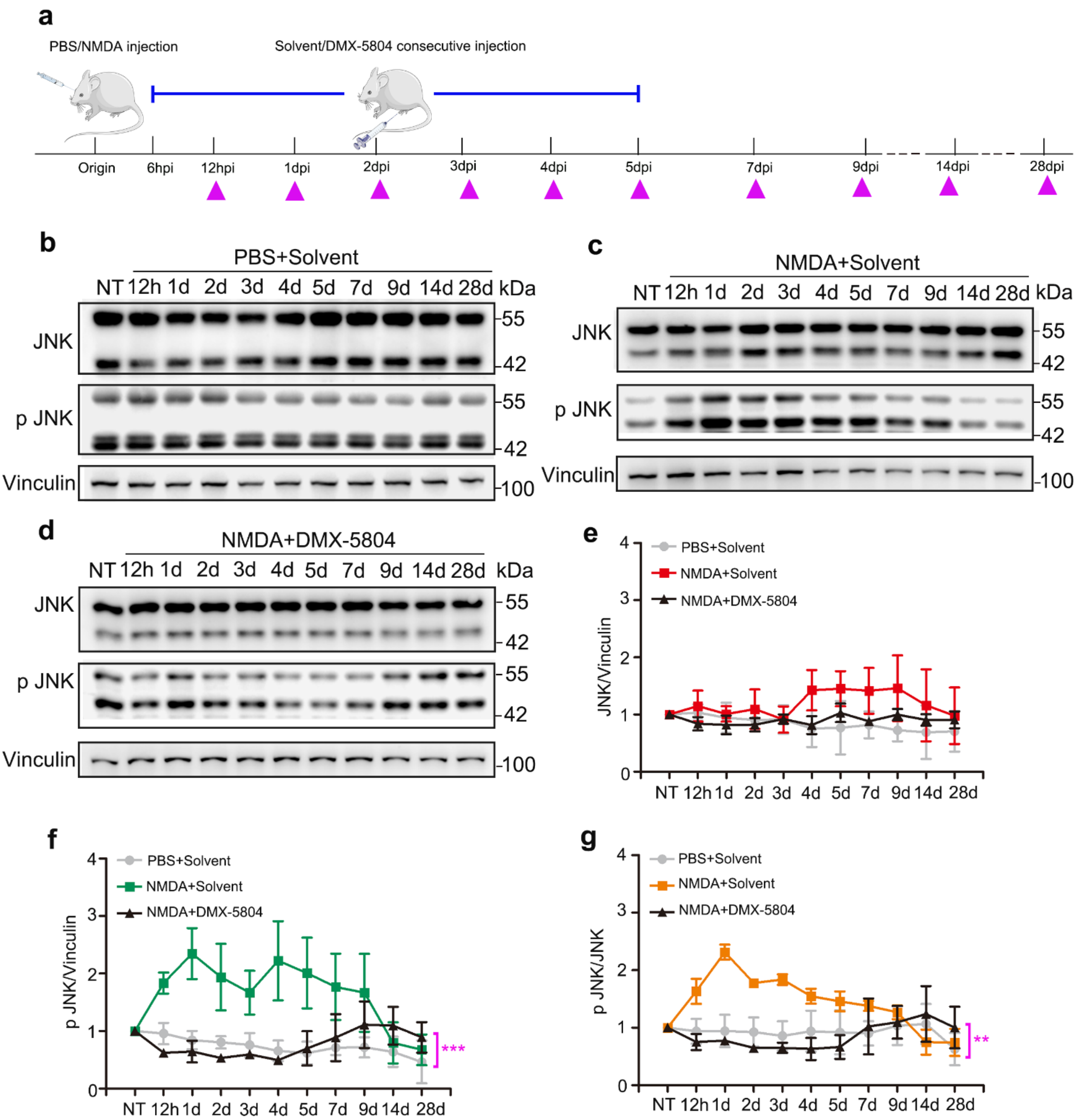

Supplementary Figure 17. DMX-5804 suppresses JNK pathway activities in the NMDA-injured retina. **a** Timeline diagram of the experimental procedures used in **b-g**. Wild type mice were intravitreally injected with PBS or NMDA. Solvent or DMX-5804 was injected starting from 6 hpi until 5 dpi with injections every 6 h. Mice were sacrificed at the indicated time points. **b-d** Western blots analysis of p JNK and JNK at indicated time points after PBS (**b**), NMDA/solvent (**c**), or NMDA/DMX-5804 (**d**) injections. The *purpl*e triangles represent the time points of the sampling. **e-g** Quantification (normalized to vinculin) of p JNK and JNK in **b-d**. For Western blots, levels were given as a.u. ± SEM in comparison with no-treatment (NT)

group (4 samples per pool; n = 3 independent pooled samples per group; one-way ANOVA test). *$p \leq 0.05$, **$p \leq 0.01$, ***$p \leq 0.001$.

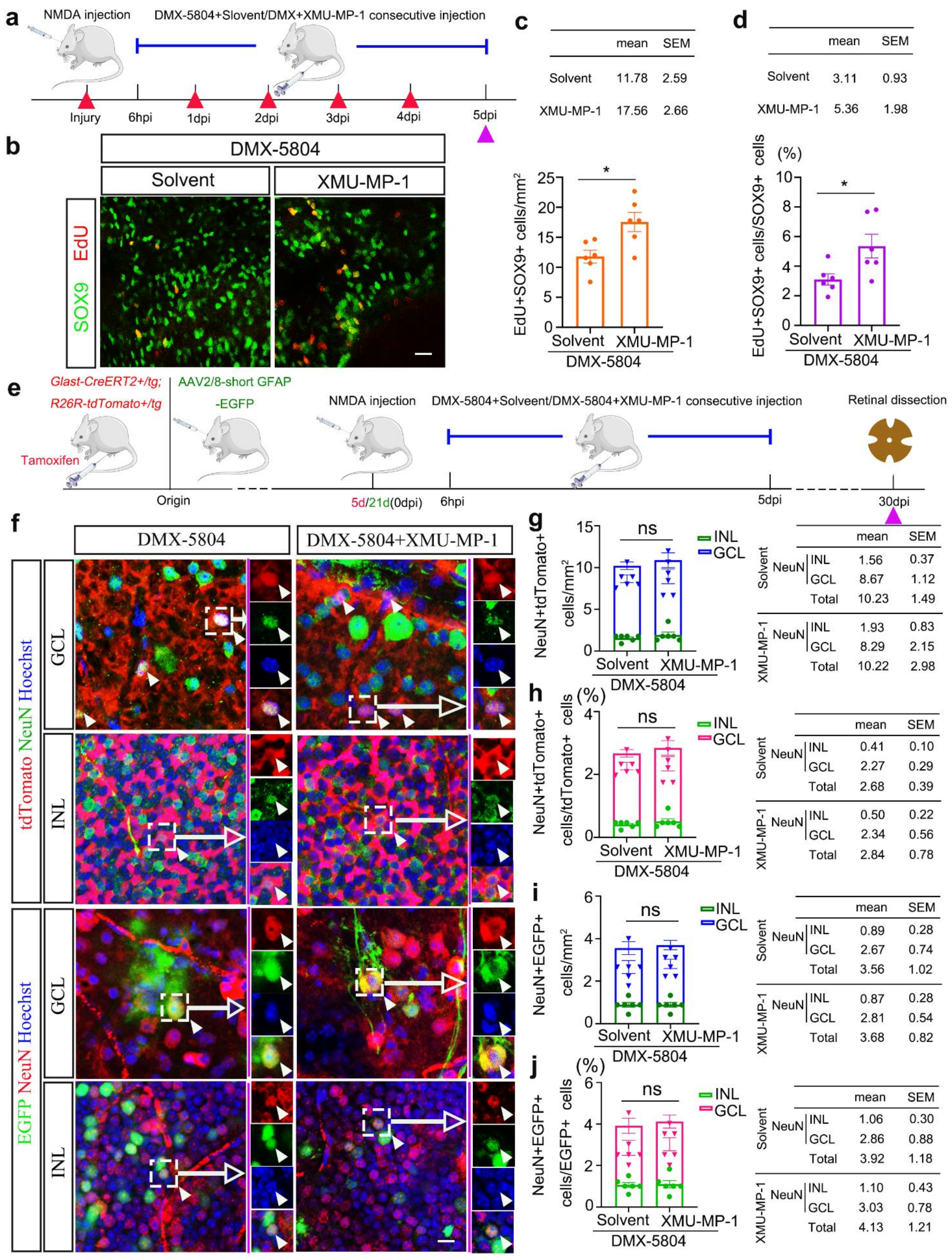

Supplementary Figure 18. The synergistic effects of DMX-5804 and XMU-MP-1. a Timeline diagram of the experimental procedures used in **b-d**. Wild type mice were intravitreally injected with NMDA first.

XMU-MP-1 or DMX-5804 was injected starting from 6 hpi until 5 dpi with injections every 6 h. EdU was injected intraperitoneally every 24 hours. Mice were sacrificed 5 days later. The red triangles represent the time points of the EdU injection and the *purple* triangles represent the time points of the sampling. **b** EdU labeling (Edu+; *red*) and SOX9 (SOX9+; *green*) immunofluorescence on whole flat-mounted retinas 5 days after NMDA injection and consecutive DMX-5804+Solvent or DMX-5804+XMU-MP-1 treatment. **c** Quantification of the number of EdU+SOX9+ cells on whole flat-mounted retinas 5 days after NMDA injection and consecutive DMX-5804+Solvent or DMX-5804+XMU-MP-1 treatment. **d** Quantification of the percentage of EdU+ SOX9+ cells in SOX9+ cells on whole flat-mounted retinas 5 days after NMDA injection and consecutive DMX-5804+Solvent or DMX-5804+XMU-MP-1 treatment. **e** Timeline diagram of the experimental procedures used in **f-j**. Two systems were used. One is Glast-CreERT2+/tg;ROSA26R-tdTomato+/tg mice were intraperitoneally injected with Tamoxifen, and the other is wild type mice were intravitreally injected with pAAV-short GFAP-MCS-EGFP-3FLAG. For Glast-CreERT2+/tg;ROSA26R-tdTomato+/tg mice which were injected with Tamoxifen, NMDA was administered into the vitreous 5 days later. For wild type mice, which were intravitreally injected with pAAV-short GFAP-MCS-EGFP-3FLAG, NMDA was administered into the vitreous 3 wk later. XMU-MP-1 or DMX-5804 was firstly injected starting from 6 hpi until 5 dpi with injections every 6 h. The *purple* triangles represent the time points of the sampling. **f** TdTomato and NeuN (NeuN+; *green*) immunofluorescence or EGFP and NeuN (NeuN+; *red*) immunofluorescence on whole flat-mounted retinas after NMDA injection and consecutive DMX-5804+Solvent or DMX-5804+XMU-MP-1 treatment. **g** Quantification of the number of tdTomato+NeuN+ cells on whole flat-mounted retinas 30 days after NMDA injection and consecutive DMX-5804+Solvent or DMX-5804+XMU-MP-1 treatment. **h** Quantification of the percentage of tdTomato+NeuN+ cells in tdTomato+ cells on whole flat-mounted retinas 5 days after NMDA injection and consecutive DMX-5804+Solvent or DMX-5804+XMU-MP-1 treatment. **i** Quantification of the number of EGFP+NeuN+ cells on whole flat-mounted retinas 30 days after NMDA injection and consecutive DMX-5804+Solvent or DMX-5804+XMU-MP-1 treatment. **j** Quantification of the percentage of EGFP+NeuN+ cells in EGFP+ cells on whole flat-mounted retinas 5 days after NMDA injection and consecutive DMX-5804+Solvent or DMX-5804+ XMU-MP-1 treatment. Scale bars, 20 μm (**b, f**). For quantification of EdU+ SOX9+, tdTomato+NeuN+ or EGFP+NeuN+ cells, levels were given as mean ± SEM (n = 6 mice per group; Student's *t*-test). *$p \le 0.05$, **$p \le 0.01$, ***$p \le 0.001$.

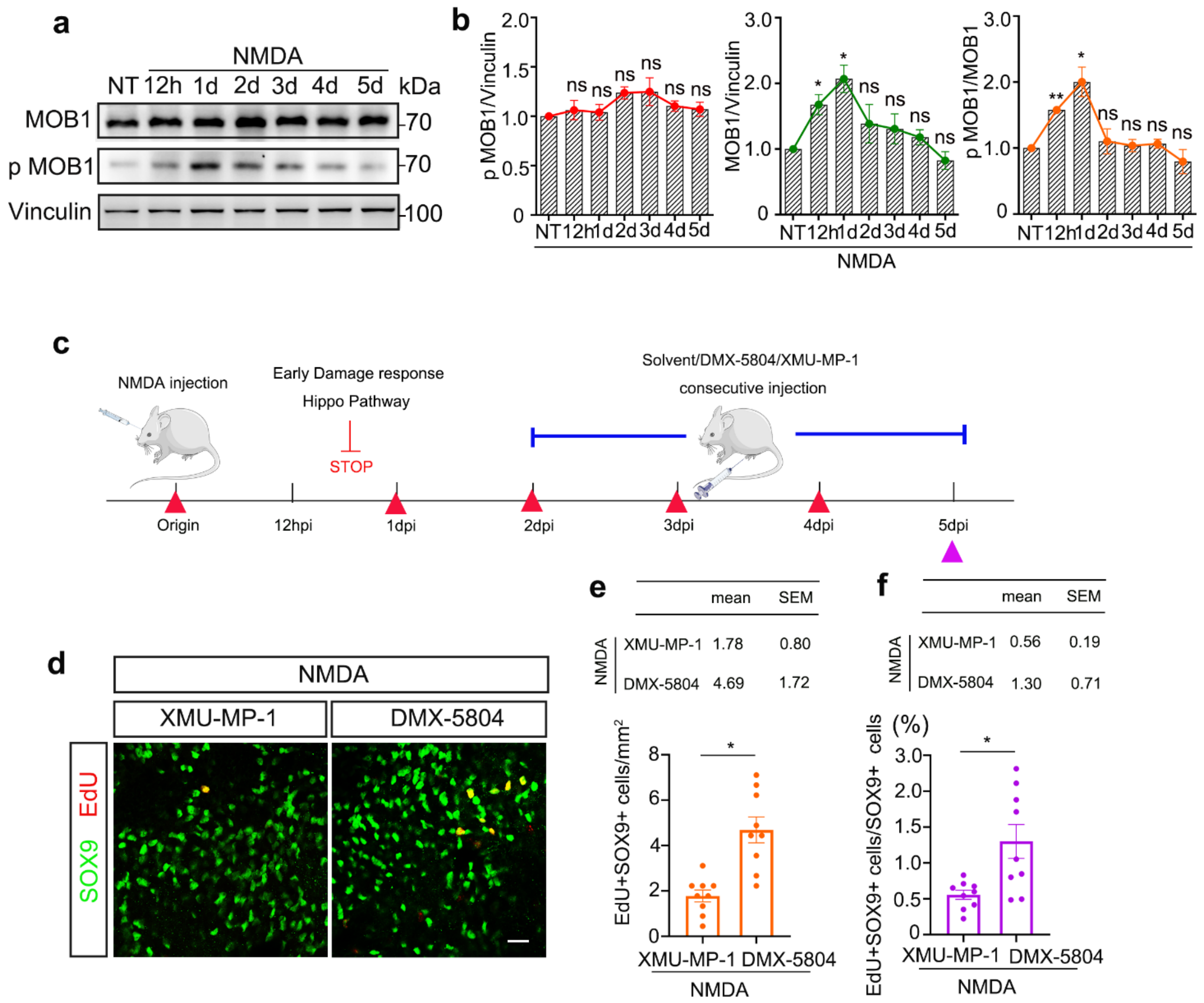

Supplementary Figure 19. DMX-5804 has higher potency than XMU-MP-1 to promote MG proliferation when drugs were treated after acute phase of retinal injury. a, b Western blots (**a**) and quantification (**b**; normalized to vinculin) of p MOB1 and MOB1 at indicated time points in the NMDA-injured retinas. **c** Timeline diagram of the experimental procedures used in **d-f**. Wild type mice were intravitreally injected with NMDA first. Next, XMU-MP-1 or DMX-5804 was injected starting from 6 hpi until 5 dpi with injections every 6 h. EdU was injected intraperitoneally every 24 h. Mice were sacrificed at day 5 post-retinal injury. The *red* triangles represent the time points of the EdU injection and the *purple* triangles represent the time points of the sampling. **d** EdU labeling (EdU+; *red*) and SOX9 (SOX9+; *green*) immunofluorescence on whole flat-mounted retinas 5 days after NMDA injection and XMU-MP-1 or DMX-5804 consecutive treatment. **e** Quantification of the number of EdU+SOX9+ cells on whole flat-mounted retinas 5 days after NMDA injection and XMU-MP-1 or DMX-5804 consecutive treatment. **f** Quantification

of the percentage of EdU+SOX9+ cells in SOX9+ cells on whole flat-mounted retinas 5 days after NMDA injection and XMU-MP-1 or DMX-5804 consecutive treatment. Scale bar, 20 μm (**d**). For Western blots, levels were given as a.u. ± SEM in comparison with no-treatment (NT) group (4 samples per pool; n = 3 independent pooled samples per group; Student's *t*-test). For quantification of EdU+ SOX9+ cells, levels were given as mean ± SEM (n = 9 mice per group; Student's *t*-test). *$p \leq 0.05$, **$p \leq 0.01$, ***$p \leq 0.001$.

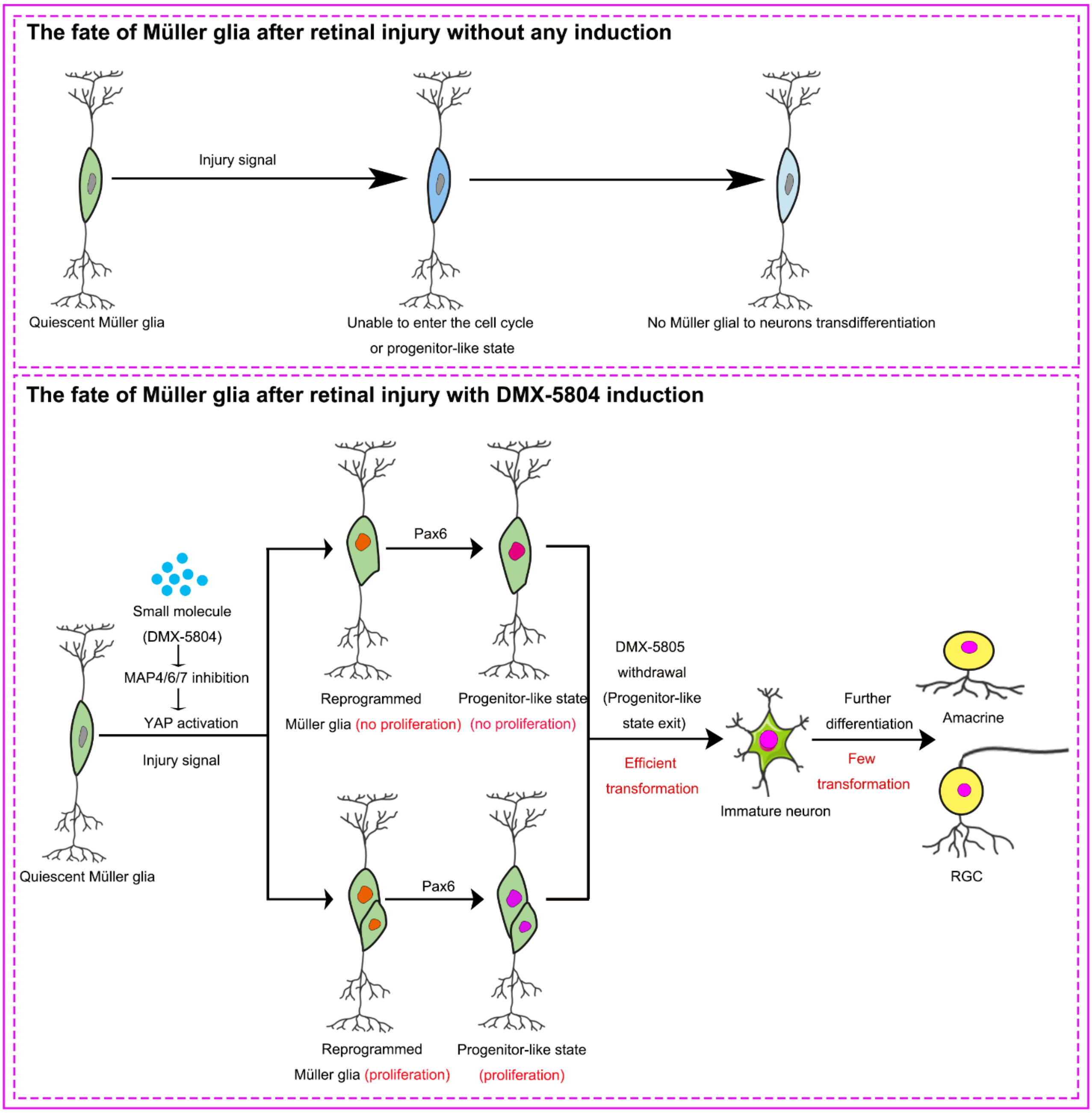

Supplementary Figure 20. Working model of MAP4Ks inhibition promoted retinal regeneration. By using a selective small-molecule inhibitor, DMX-5804, which simultaneously inhibits MAP4K4, MAP4K6 and MAP4K7 to activate YAP in the retina, MGs are able to reprogram and transdifferentiate into retinal neurons expressing both amacrine and RGC markers after retinal damage in adult mice. The elements contained in this scheme were created by Houjian Zhang.

Figure 1

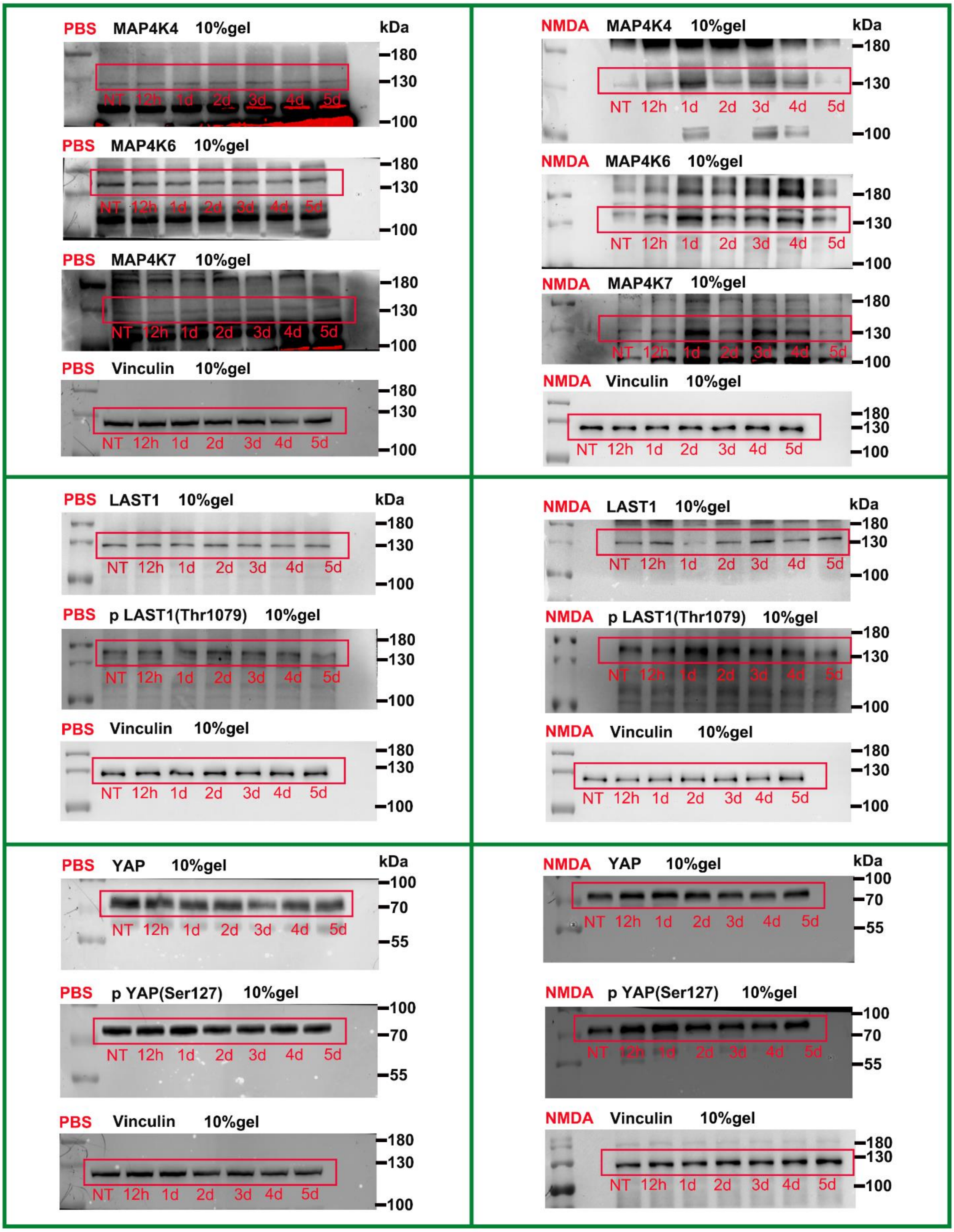
PBS MAP4K4 10%gel
kDa
180
130
100
NT 12h 1d 2d 3d 4d 5d
PBS MAP4K6 10%gel
PBS MAP4K7 10%gel
PBS Vinculin 10%gel
NMDA MAP4K4 10%gel
NMDA MAP4K6 10%gel
NMDA MAP4K7 10%gel
NMDA Vinculin 10%gel
PBS LAST1 10%gel
PBS p LAST1(Thr1079) 10%gel
PBS Vinculin 10%gel
NMDA LAST1 10%gel
NMDA p LAST1(Thr1079) 10%gel
NMDA Vinculin 10%gel
PBS YAP 10%gel
PBS p YAP(Ser127) 10%gel
PBS Vinculin 10%gel
NMDA YAP 10%gel
NMDA p YAP(Ser127) 10%gel
NMDA Vinculin 10%gel
70
55

Figure 2

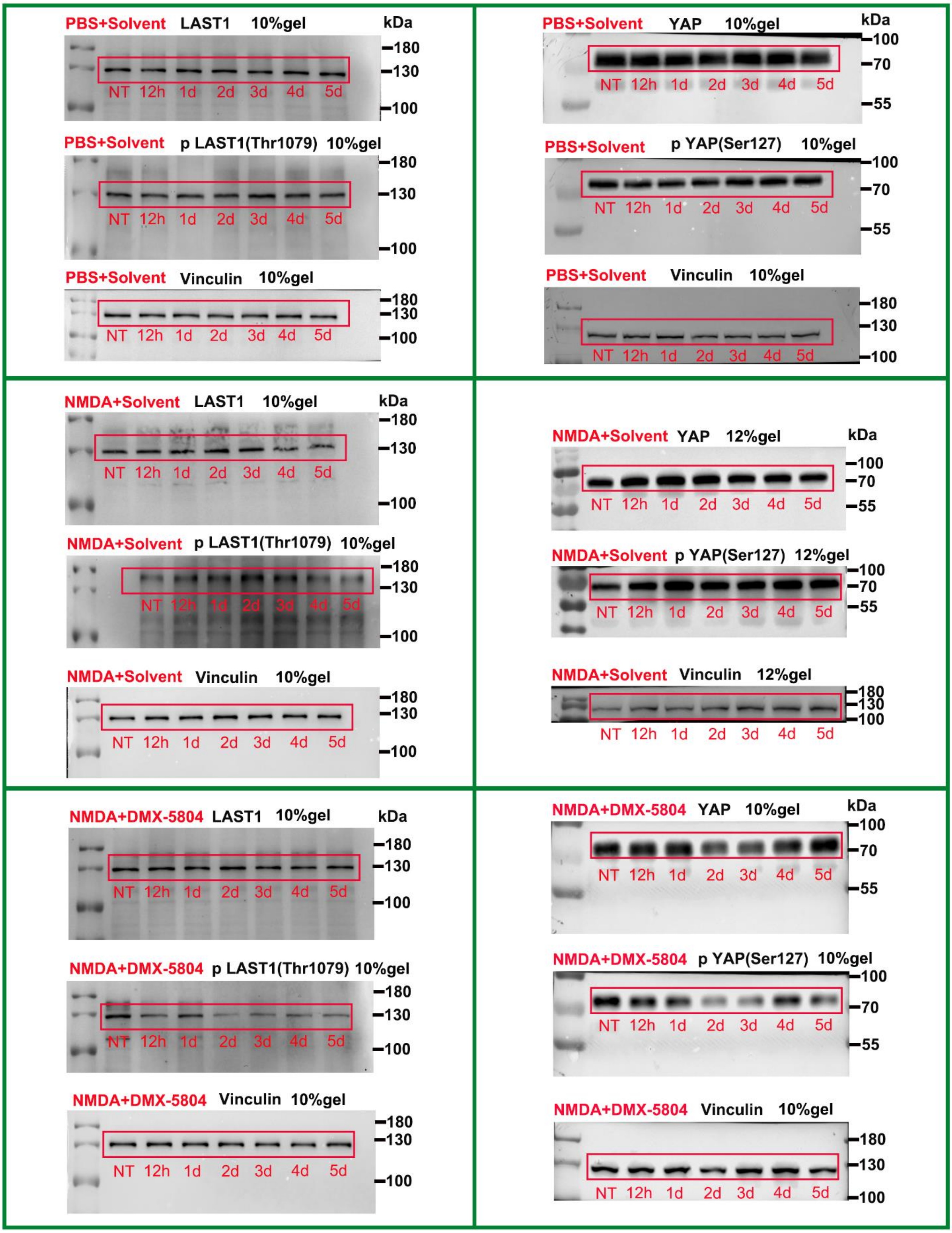
PBS+Solvent LAST1 10%gel
kDa
180
130
NT 12h 1d 2d 3d 4d 5d
100
PBS+Solvent p LAST1(Thr1079) 10%gel
PBS+Solvent Vinculin 10%gel
PBS+Solvent YAP 10%gel
100
70
55
PBS+Solvent p YAP(Ser127) 10%gel
PBS+Solvent Vinculin 10%gel
NMDA+Solvent LAST1 10%gel
NMDA+Solvent p LAST1(Thr1079) 10%gel
NMDA+Solvent Vinculin 10%gel
NMDA+Solvent YAP 12%gel
NMDA+Solvent p YAP(Ser127) 12%gel
NMDA+Solvent Vinculin 12%gel
NMDA+DMX-5804 LAST1 10%gel
NMDA+DMX-5804 p LAST1(Thr1079) 10%gel
NMDA+DMX-5804 Vinculin 10%gel
NMDA+DMX-5804 YAP 10%gel
NMDA+DMX-5804 p YAP(Ser127) 10%gel
NMDA+DMX-5804 Vinculin 10%gel

Supplementary Figure 1

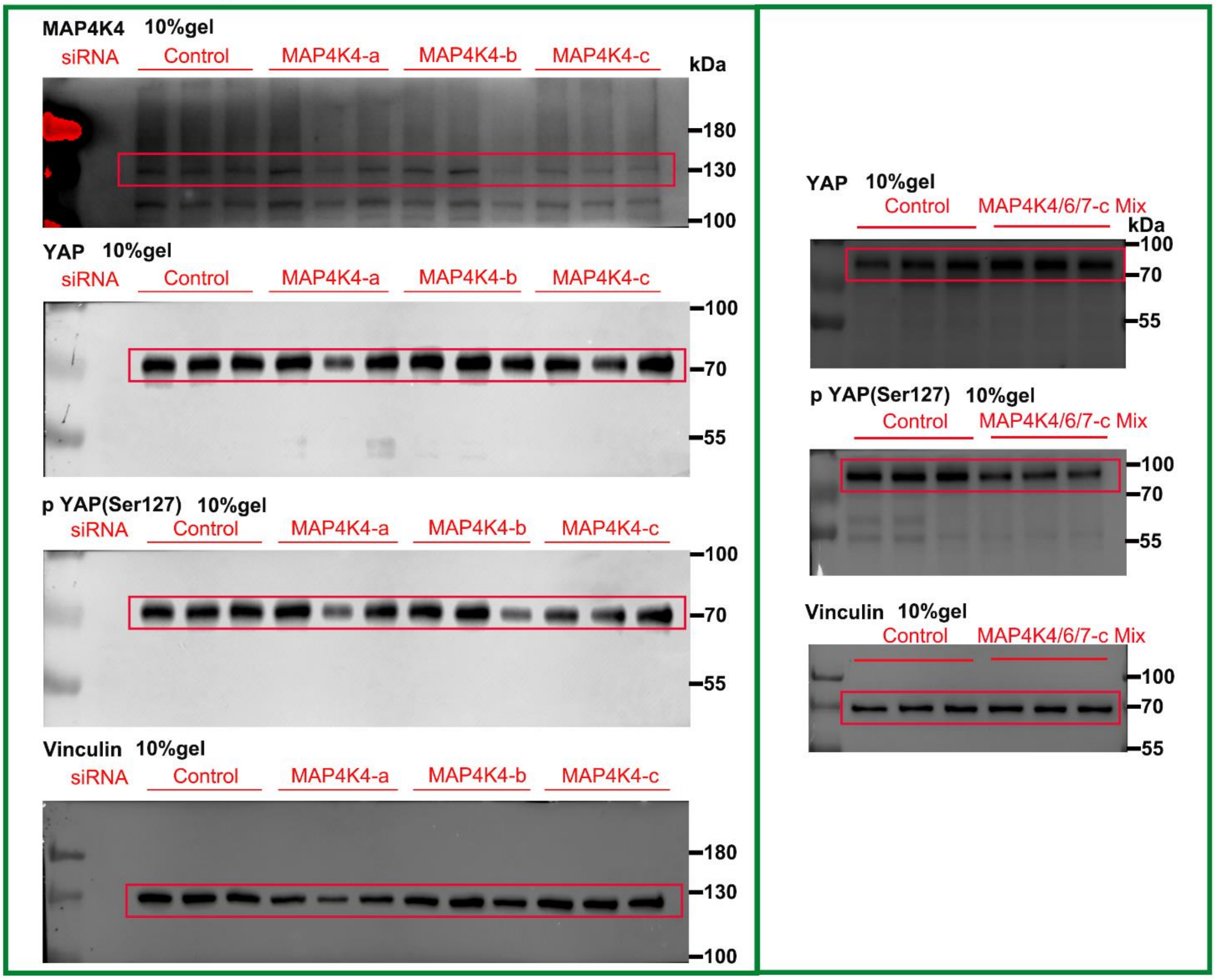

Supplementary Figure 3

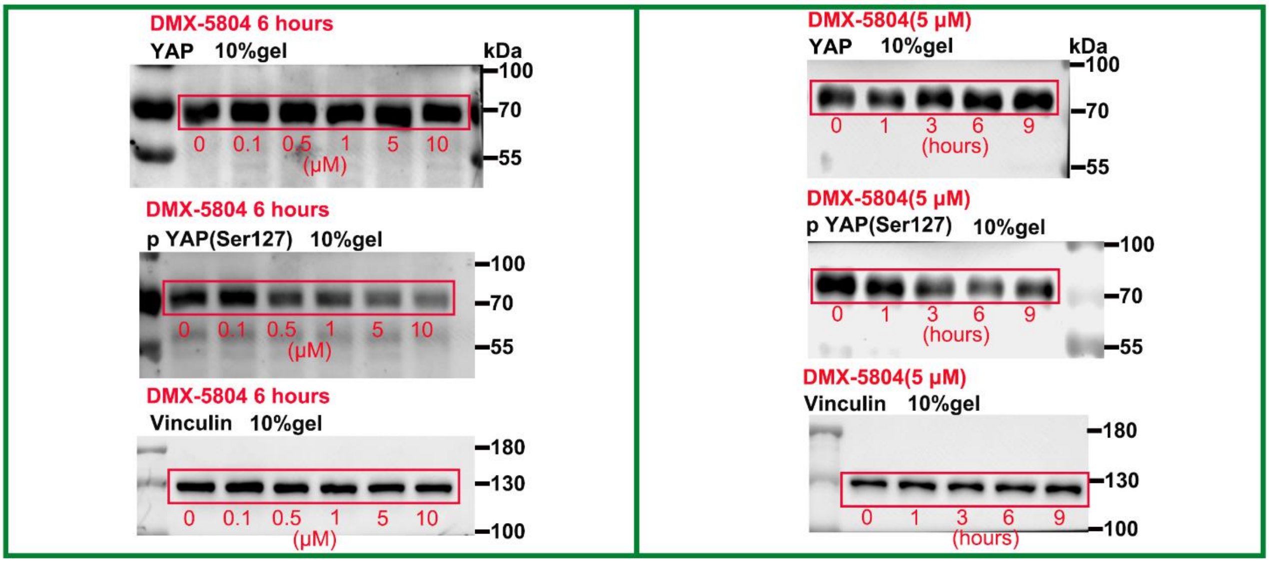

Supplementary Figure 4

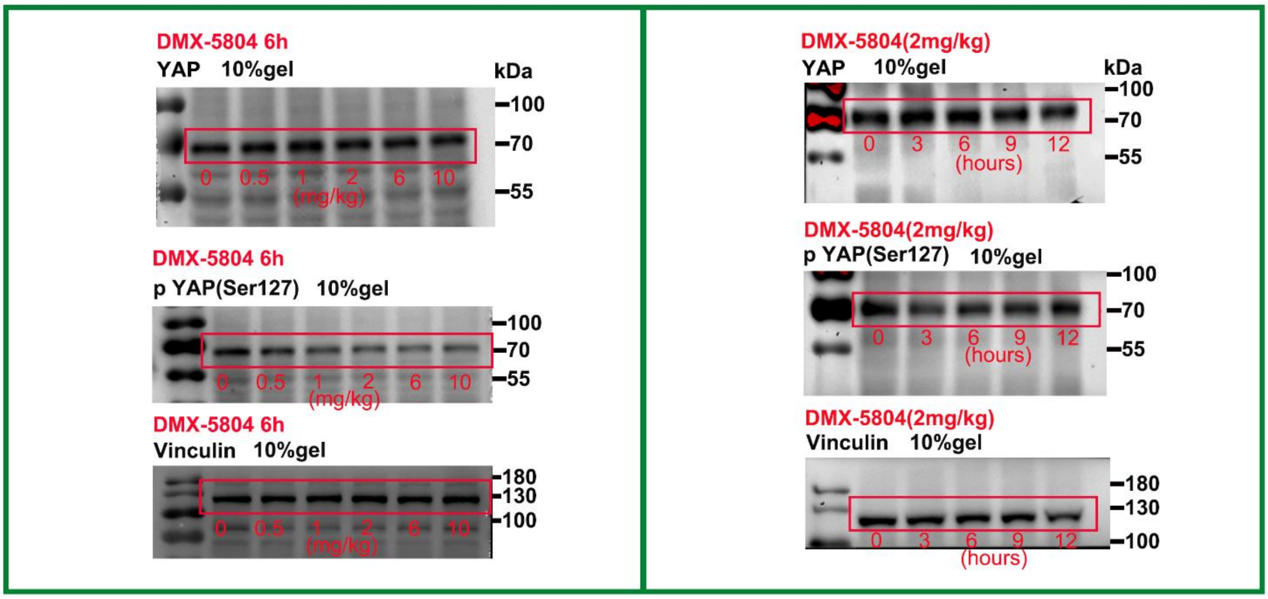

Supplementary Figure 14

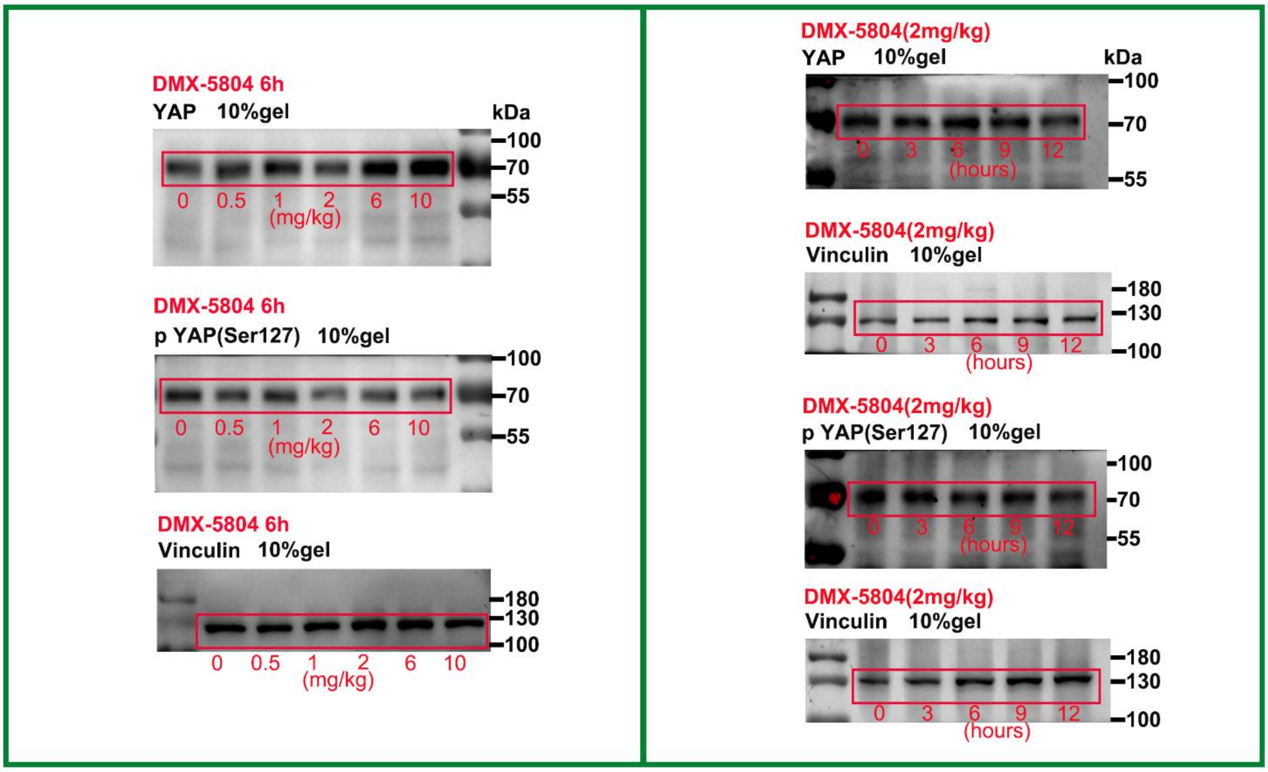

Supplementary Figure 16

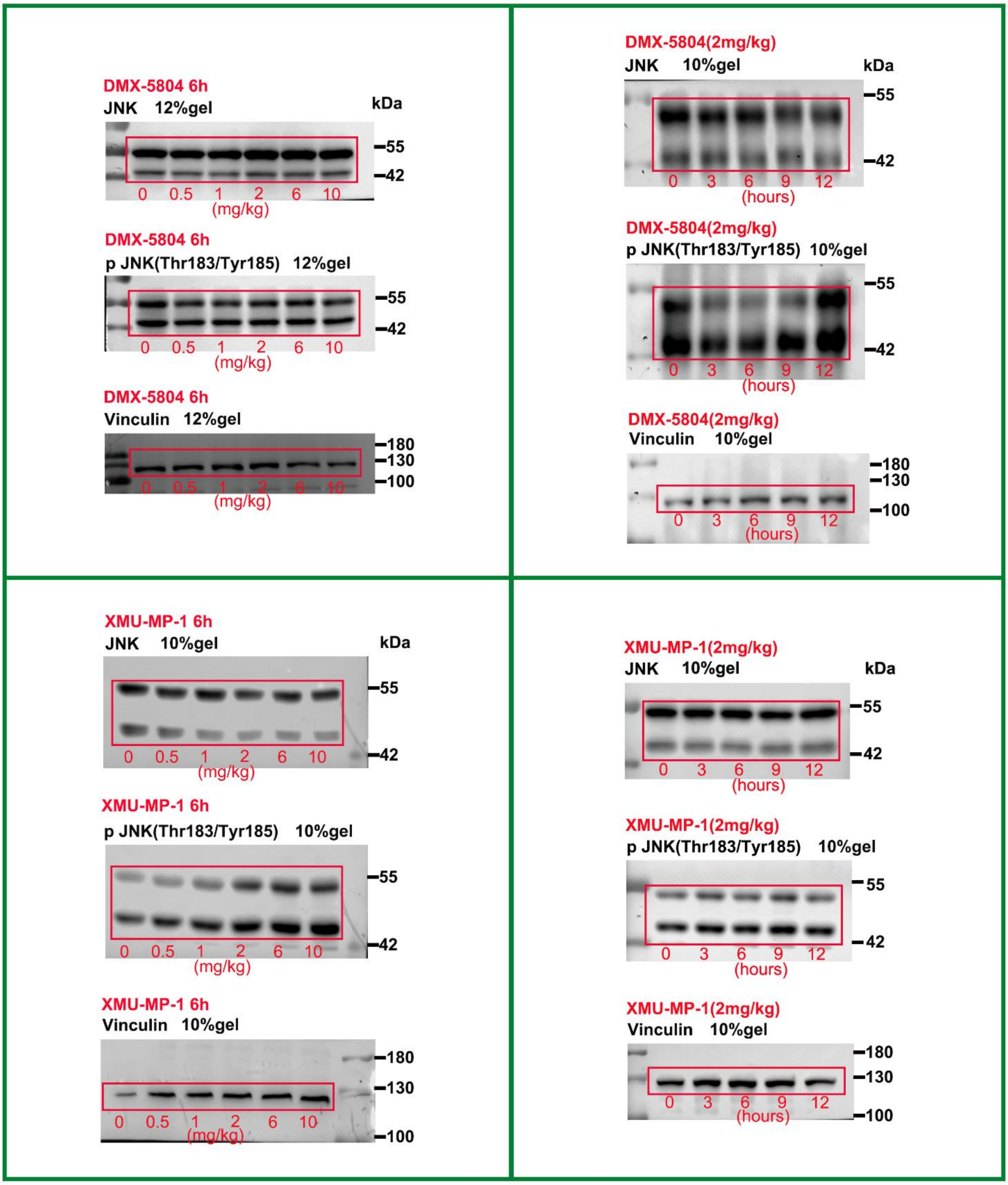

Supplementary Figure 17

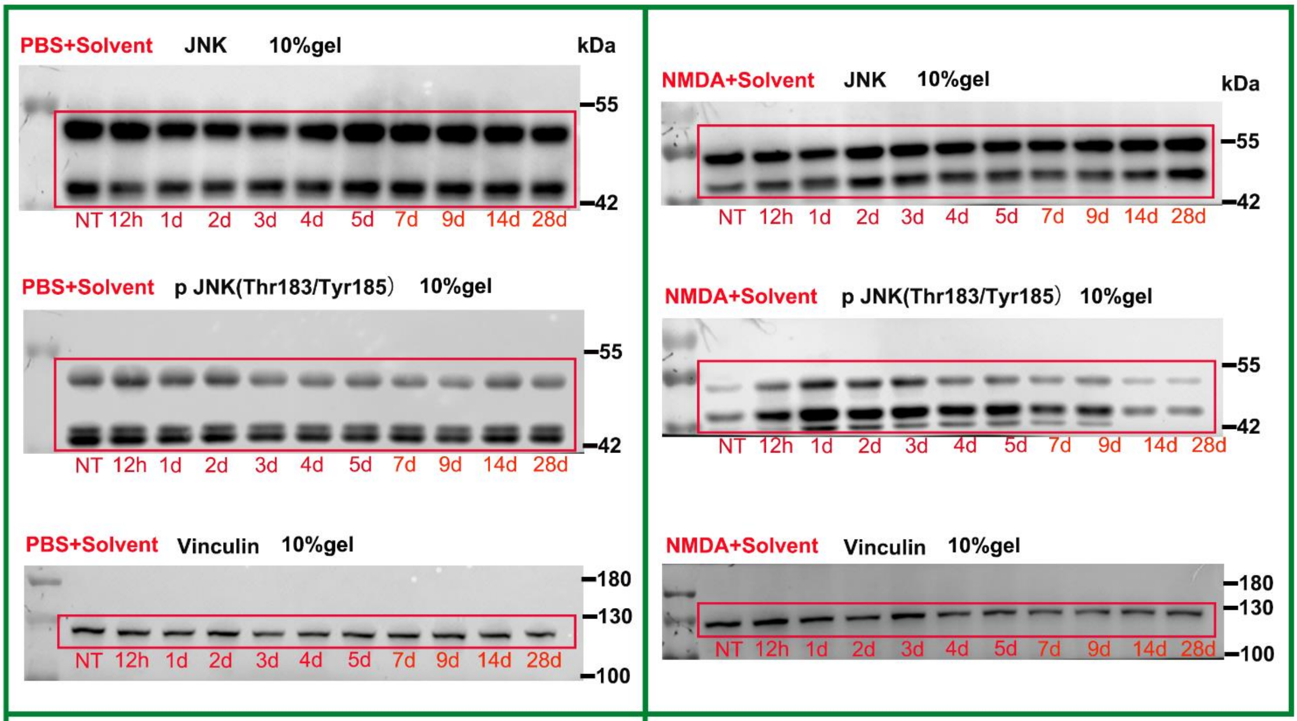

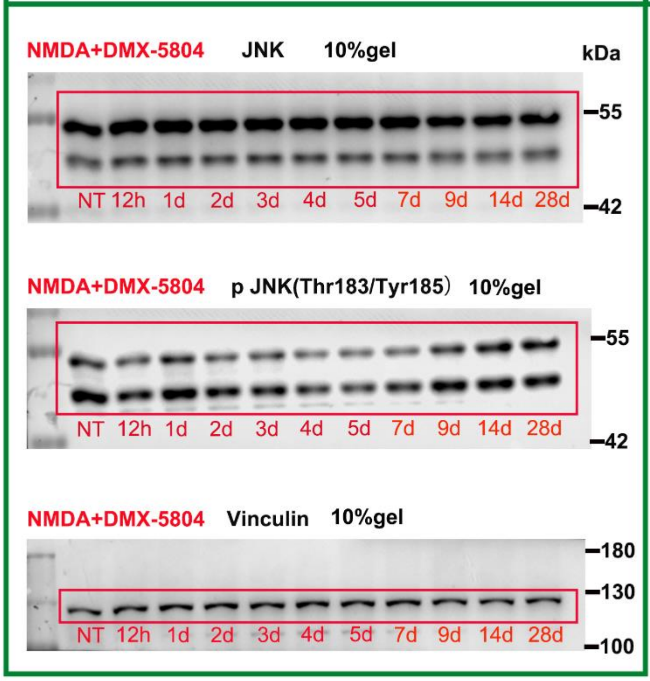

Supplementary Figure 19

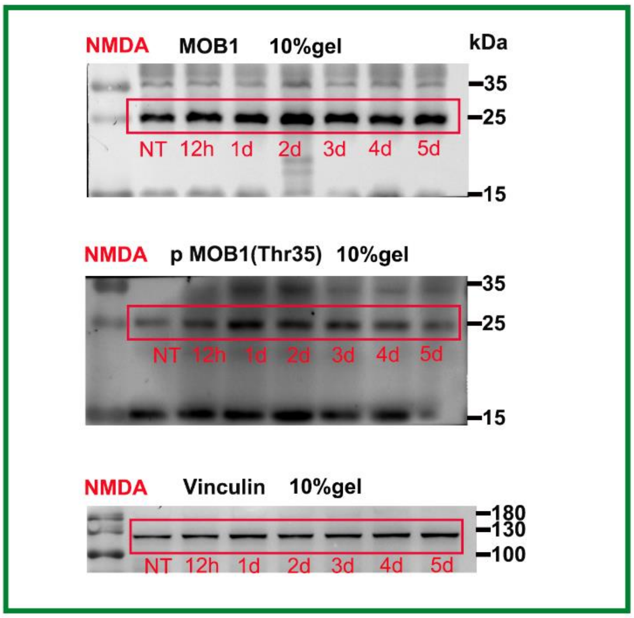

Supplementary Figure 21. Uncropped images of Western blots. Uncropped scans of the most important blots including Figure 1, Figure 2, Supplementary Figure 1, Supplementary Figure 3, Supplementary Figure 4, Supplementary Figure 14, Supplementary Figure 16, Supplementary Figure 17 and Supplementary Figure 19. Red boxes indicate areas that are cropped.
