# Supplementary material for: MAP4Ks inhibition promotes retinal neuron regeneration from Müller glia in adult mice
Source: NPJ Regen Med. 2023 Jul 13;8:36. doi: 10.1038/s41536-023-00310-6 (PMC10344969; doi:10.1038/s41536-023-00310-6)
Supplement: Supplementary file 2 — Reporting Summary [file 41536_2023_310_MOESM2_ESM.pdf]

nature portfolio

Corresponding author(s): Yi Liao, Zuguo Liu

Last updated by author(s): Jun 25, 2023

Reporting Summary

Nature Portfolio wishes to improve the reproducibility of the work that we publish. This form provides structure for consistency and transparency in reporting. For further information on Nature Portfolio policies, see our Editorial Policies and the Editorial Policy Checklist.

Statistics

For all statistical analyses, confirm that the following items are present in the figure legend, table legend, main text, or Methods section.

n/a	Confirmed	
☐	☒	The exact sample size (*n*) for each experimental group/condition, given as a discrete number and unit of measurement
☐	☒	A statement on whether measurements were taken from distinct samples or whether the same sample was measured repeatedly
☐	☒	The statistical test(s) used AND whether they are one- or two-sided<br>*Only common tests should be described solely by name; describe more complex techniques in the Methods section.*
☒	☐	A description of all covariates tested
☒	☐	A description of any assumptions or corrections, such as tests of normality and adjustment for multiple comparisons
☐	☒	A full description of the statistical parameters including central tendency (e.g. means) or other basic estimates (e.g. regression coefficient) AND variation (e.g. standard deviation) or associated estimates of uncertainty (e.g. confidence intervals)
☐	☒	For null hypothesis testing, the test statistic (e.g. F, t, r) with confidence intervals, effect sizes, degrees of freedom and P value noted<br>*Give P values as exact values whenever suitable.*
☒	☐	For Bayesian analysis, information on the choice of priors and Markov chain Monte Carlo settings
☒	☐	For hierarchical and complex designs, identification of the appropriate level for tests and full reporting of outcomes
☐	☒	Estimates of effect sizes (e.g. Cohen's *d*, Pearson's *r*), indicating how they were calculated

Our web collection on statistics for biologists contains articles on many of the points above.

Software and code

Policy information about availability of computer code

Data collection	Image LabTM software (Bio-Rad Laboratories Inc, Hercules, CA, USA), ZEN2.3 lite software (Carl Zeiss IMT Co. Ltd. Germany)
Data analysis	GraphPad Prism version 9.0 software (GraphPad Software Inc., San Diego, CA, USA)

For manuscripts utilizing custom algorithms or software that are central to the research but not yet described in published literature, software must be made available to editors and reviewers. We strongly encourage code deposition in a community repository (e.g. GitHub). See the Nature Portfolio guidelines for submitting code & software for further information.

Data

Policy information about availability of data

All manuscripts must include a data availability statement. This statement should provide the following information, where applicable:

- Accession codes, unique identifiers, or web links for publicly available datasets
- A description of any restrictions on data availability
- For clinical datasets or third party data, please ensure that the statement adheres to our policy

All data supporting the conclusions of this study are either provided in this published paper (and its Supplementary Information files) or available from the authors upon reasonable request.

Human research participants

Policy information about studies involving human research participants and Sex and Gender in Research.

Reporting on sex and gender	No human research participants
Population characteristics	No human research participants
Recruitment	No human research participants
Ethics oversight	No human research participants

Note that full information on the approval of the study protocol must also be provided in the manuscript.

Field-specific reporting

Please select the one below that is the best fit for your research. If you are not sure, read the appropriate sections before making your selection.

☒ Life sciences ☐ Behavioural & social sciences ☐ Ecological, evolutionary & environmental sciences

For a reference copy of the document with all sections, see nature.com/documents/nr-reporting-summary-flat.pdf

Life sciences study design

All studies must disclose on these points even when the disclosure is negative.

Sample size	For Western blot, 4 entire retinas of adult mice were collected for per sample pool which has been proved to be sufficient to assess the level of various proteins expression. For the assessment on whole flat-mounted, the entire retina of an adult mice were collected for per repeat.
Data exclusions	Some PhNR waves were excluded according to ISCEV guidelines.
Replication	We have increased the number of mice in each experiment to equal or more than 6 and confirmed the replications were successful.
Randomization	Both male and female adult mice were used with a randomization for setting up groups in experiments.
Blinding	This is not a clinical trial for new drug development, so blinding was not relevant to our study.

Reporting for specific materials, systems and methods

We require information from authors about some types of materials, experimental systems and methods used in many studies. Here, indicate whether each material, system or method listed is relevant to your study. If you are not sure if a list item applies to your research, read the appropriate section before selecting a response.

Materials & experimental systems

n/a	Involved in the study	
☐	☒	Antibodies
☐	☒	Eukaryotic cell lines
☒	☐	Palaeontology and archaeology
☐	☒	Animals and other organisms
☒	☐	Clinical data
☒	☐	Dual use research of concern

Methods

n/a	Involved in the study	
☒	☐	ChIP-seq
☒	☐	Flow cytometry
☒	☐	MRI-based neuroimaging

Antibodies

Antibodies used	YAP (CST, apply to Western blot, mAb, Cat. No. 14074, Lot. No. 4), p YAP (CST, apply to Western blot, mAb, Cat. No. 13008, Lot. No. 4), LATS1 (CST, apply to Western blot, mAb, Cat. No. #3477, Lot. No. 7), Phospho-LATS1 (CST, apply to Western blot, mAb, Cat. No. #8654, Lot. No. 7), MAP4K4 (CST,apply to Western blot and immunofluorescence, pAb, Cat. No. #3485, Lot. No. 4), MAP4K6 (Novus, apply to Western blot and immunofluorescence, pAb, Cat. No. NBP1-22989, Lot. No. A1) , MAP4K7 (Genetex, apply to Western blot and immunofluorescence, pAb, Cat. No. GTX13141, Lot. No. 41037), JNK (CST, apply to Western blot, Cat. No. #9252), Phospho-JNK (CST, apply to Western blot, Cat. No. #4668), MOB1 (CST, apply to Western blot, mAb, Cat. No. 13730, Lot. No. 2), p MOB1 (CST, apply to Western blot, mAb, Cat. No. #8699, Lot. No. 2). Vinculin (CST, apply to Western blot, mAb, Cat. No. 13901, Lot. No. 7),

	Glutamine Synthetase (Millipore, apply to immunofluorescence, mAb, Cat. No. MABN1182), GFAP (Millipore, apply to immunofluorescence, mAb, Cat. No. G6171), ki67 (Abcam, apply to immunofluorescence, mAb, Cat. No. ab16667, Lot.No. GR3375640-35), PCNA (CST, apply to immunofluorescence, mAb, Cat. No. #13110, Lot. No. 4), HuC/D (Abcam, apply to immunofluorescence, mAb, Cat. No. ab184267). YAP (Santa Cruz, apply to immunofluorescence, mAb, Cat. No. 101199, Lot. No. #H2322), SOX9 (Abcam, apply to immunofluorescence, mAb, Cat. No. ab185966, Lot. No. 4), Pax6 (Santa Cruz, apply to immunofluorescence, mAb, Cat. No. sc81649, Lot. No. 4), Pax6 (Abcam, apply to immunofluorescence, mAb, Cat. No. ab195045, Lot. No. 1016697-4), NeuN (Novus, apply to immunofluorescence, mAb, Cat. No. NBP1-92693, Lot. No. #G2420), NeuN (Abcam, apply to immunofluorescence, mAb, Cat. No. ab177487, Lot. No. GR3275122-26), GAD67 (Abcam, apply to immunofluorescence, mAb, Cat. No. ab26116, Lot. No. GR3423991-1), RBPMS (Proteintech, apply to immunofluorescence, mAb, Cat. No. 15187-1-AP, Lot. No. 00080064), β-tubulin (Abcam, apply to immunofluorescence, mAb, Cat. No. ab18207, Lot. No. GR3452682-3), Recoverin (Proteintech, apply to immunofluorescence, mAb, Cat. No. 10073-1-AP), Rhodopsin (ABclonal, apply to immunofluorescence, mAb, Cat. No. A7245), Arrestin (Millipore, apply to immunofluorescence, mAb, Cat. No. AB15282), Calretinin (Abcam, apply to immunofluorescence, mAb, Cat. No. ab92341, Lot. No. GR38060-26), MAP2 (Abcam, apply to immunofluorescence, mAb, Cat. No. ab183830, Lot. No. 1023498-3),
Validation	All primary antibodies has been validated on mice and cited by other manuscripts.

Eukaryotic cell lines

Policy information about cell lines and Sex and Gender in Research

Cell line source(s)	MIO-M1cells (RRID: CVCL_0433) were a kind gift from Wenzhou Medical University. The primary Müller glia cells were isolated from adult mice without sex distinction.
Authentication	MIO-M1 cells were authenticated by Müller glia markers labeling.
Mycoplasma contamination	The MIO-M1 cell line was not tested for mycoplasma contamination.
Commonly misidentified lines (See ICLAC register)	No commonly misidentified lines was used in our study.

Animals and other research organisms

Policy information about studies involving animals; ARRIVE guidelines recommended for reporting animal research, and Sex and Gender in Research

Laboratory animals	C57BL/6J mice were use for the retinal injury model and these mice were at least 40 days old and no more than 70 days.
Wild animals	The study did not involve wild animals.
Reporting on sex	All experiments involving adult mice were performed with male or female mice. No difference between sexes was observed in any retinal phenotype.
Field-collected samples	The study did not involve samples collected from the field.
Ethics oversight	All animals were housed in Xiamen University Laboratory Animal Center; all procedures were approved by the Xiamen University Institutional Animal Care and Use Committee (IACUC) and complied with the ARVO Statement for the Use of Animals in Ophthalmic and Vision Research.

Note that full information on the approval of the study protocol must also be provided in the manuscript.
